# Analysis of LAGEs Family Gene Signature and Prognostic Relevance in Breast Cancer

**DOI:** 10.3390/diagnostics11040726

**Published:** 2021-04-19

**Authors:** Hoang Dang Khoa Ta, Wan-Chun Tang, Nam Nhut Phan, Gangga Anuraga, Sz-Ying Hou, Chung-Chieh Chiao, Yen-Hsi Liu, Yung-Fu Wu, Kuen-Haur Lee, Chih-Yang Wang

**Affiliations:** 1 PhD Program for Cancer Molecular Biology and Drug Discovery, College of Medical Science and Technology, Taipei Medical University and Academia Sinica, Taipei 11031, Taiwan; d621109004@tmu.edu.tw (H.D.K.T.); g.anuraga@unipasby.ac.id (G.A.); 2 Graduate Institute of Cancer Biology and Drug Discovery, College of Medical Science and Technology, Taipei Medical University, Taipei 11031, Taiwan; yeas0310@hotmail.com (W.-C.T.); syhoutmu@tmu.edu.tw (S.-Y.H.); 3 NTT Institute of Hi-Technology, Nguyen Tat Thanh University, Ho Chi Minh City 700000, Vietnam; pnnam@ntt.edu.vn; 4 Department of Statistics, Faculty of Science and Technology, Universitas PGRI Adi Buana, Surabaya 60234, East Java, Indonesia; 5 School of Chinese Medicine for Post-Baccalaureate, I-Shou University, Kaohsiung 82445, Taiwan; isu10556045a@cloud.isu.edu.tw (C.-C.C.); isu10556037a@cloud.isu.edu.tw (Y.-H.L.); 6 Department of Medical Research, Tri-Service General Hospital, School of Medicine, National Defense Medical Center, Taipei 11490, Taiwan; qrince@yahoo.com.tw; 7 Cancer Center, Wan Fang Hospital, Taipei Medical University, Taipei 11031, Taiwan

**Keywords:** bioinformatics, breast cancer, LAGE1, LAGE2A, LAGE2B, LAGE3

## Abstract

Breast cancer (BRCA) is one of the most complex diseases and involves several biological processes. Members of the L-antigen (LAGE) family participate in the development of various cancers, but their expressions and prognostic values in breast cancer remain to be clarified. High-throughput methods for exploring disease progression mechanisms might play a pivotal role in the improvement of novel therapeutics. Therefore, gene expression profiles and clinical data of LAGE family members were acquired from the cBioportal database, followed by verification using the Oncomine and The Cancer Genome Atlas (TCGA) databases. In addition, the Kaplan-Meier method was applied to explore correlations between expressions of LAGE family members and prognoses of breast cancer patients. MetaCore, GlueGo, and GluePedia were used to comprehensively study the transcript expression signatures of LAGEs and their co-expressed genes together with LAGE-related signal transduction pathways in BRCA. The result indicated that higher LAGE3 messenger (m)RNA expressions were observed in BRCA tissues than in normal tissues, and they were also associated with the stage of BRCA patients. Kaplan-Meier plots showed that overexpression of LAGE1, LAGE2A, LAGE2B, and LAGE3 were highly correlated to poor survival in most types of breast cancer. Significant associations of LAGE family genes were correlated with the cell cycle, focal adhesion, and extracellular matrix (ECM) receptor interactions as indicated by functional enrichment analyses. Collectively, LAGE family members’ gene expression levels were related to adverse clinicopathological factors and prognoses of BRCA patients; therefore, LAGEs have the potential to serve as prognosticators of BRCA patients.

## 1. Introduction

The World Health Organization (WHO) has provided statistical reports showing that cancer is one of the leading causes of premature death, and patient numbers are increasing due to an aging population [1]. Breast cancer (BRCA) accounts for the largest proportion of cancer cases in females, and exploring its progression mechanisms is crucial for therapeutic development [2,3,4,5,6]. Recently, researchers have produced rapid advances in the field of cancer therapy, where genetic alterations and the dysfunction of signal transduction pathways play important roles [7,8,9].

L-Antigen 1 (LAGE1), also known as CTAG2 (cancer/testis antigen 2) and two of its isoforms LAGE2A/CTAG1A (cancer/testis antigen 1A) and LAGE2B/CTAG2B (cancer/testis antigen 1B), as well as L-antigen family member 3 (LAGE3), are known to be part of the EKC/KEOPS complex [10,11]. LAGE family members play significant roles in RNA polymerase II-mediated regulation as well as the transfer (t)RNA threonyl-carbamoyl adenosine metabolic pathway. LAGE family members participate in cellular transformation and potentially mediate the onset of cancers. In particular, high LAGE3 expression was found in human colon, lung, and kidney tumors. In addition, LAGE family members were shown to be essential genes involved in the metastasis of primary solid tumors [12,13]. Upregulation of LAGE1 messenger (m)RNA was found in liver cancer and was associated with sarcomas [14]. Intriguingly, low LAGE3 expression was observed in several cancer tumor types, such as lung, breast, liver, and colon tumors, but not pituitary tumors [15,16]. The mRNA levels of other LAGE family members, i.e., LAGE2A and LAGE2B, were only found to be expressed in lung tumors [17,18] with lymph node and distant metastases. Many alterations in essential enzyme activities in cancers were reported that disrupt cellular metabolic pathways [19,20]. However, it is still unclear whether the immune and inflammation-related pathway were correlated with the LAGE family in BRCA.

Due to their expeditiousness and robustness in screening potential targets in diseases, high-throughput technologies are now playing critical roles in the biomedical fields. Various publicly available databases, including Gene Expression Omnibus (GEO), which covers more than 94,000 datasets and over 2 million samples, are widely employed in cancer research for both raw and processed dataset analyses [21,22,23,24,25,26]. Oncomine is mainly applied to numerous cancer types and subtypes to query mRNA gene expression profiles, and it offers multiple types of analysis, allowing researchers to compare variations in gene expressions between matched tumor and normal tissues [27,28]. Since altered gene expressions have long been proposed to play a part in cancer development as oncogenes or tumor suppressors, there is a need to understand the different perceptions of LAGE family members between various subtypes of BRCA. To our advantage, databases for LAGE functions and networks as well as gene enrichment pathways have been explored using MetaCore, a high-quality biological platform including integrated pathway and network analyses for multi-omics data [29]. To confirm the prognostic power of these family of genes, we conducted integrative data analyses based on the Kaplan-Meier database to explore the relevance of expressions of LAGE family genes as well as their prognostic levels in clinical patients [30]. In this study, the LAGE family’s functions in BRCA tumorigenesis in terms of the immune response are systematically discussed for the first time.

## 2. Materials and Methods

### 2.1. Oncomine Analysis

ONCOMINE (https://www.oncomine.org/, accessed on 30 November 2020), an online platform with a public cancer microarray database, was used to measure mRNA expression levels of the LAGE family in several cancers [27]. The mRNA expressions of the LAGE family in cancerous tissues were compared to normal tissues in BRCA patients using an independent sample t-test. Thresholds used in this analysis were as follows: fold change > 2, *p* value of <0.05, and top gene rank 10%.

### 2.2. Cancer Cell Line Encyclopedia (CCLE) Analysis

We determined expressions of LAGE family genes in various cancer types using the CCLE database [31,32], an online database that provides public access to genome data, analysis, and visualization of 947 human cancer cell lines. Expression levels were exported and charted as a heat map with a log2 transformation of expression values.

### 2.3. Functional Enrichment Analysis

We used data from the METABRIC and TCGA datasets in the cbioportal (https://www.cbioportal.org, accessed on 30 November 2020) database for functional enrichment analysis [33,34,35]. There were two parts to this analysis. First, it was used to determine if LAGE family members are highly correlated with metastatic markers. This was accomplished by searching the LAGE gene family’s expression list using Venny vers. 2.1 and then doing a Cytoscape analysis [36,37,38,39,40]. The second part determined the biological processes, disease biomarker networks, and breast neoplasm cell-cell signaling pathways using the MetaCore analysis (https://portal.genego.com, accessed on 30 November 2020). Furthermore, a gene ontology (GO) analysis was also implemented to describe genes and gene products from three categories: cell composition, molecular function, and biological processes [41,42,43]. The source database for integrating and interpreting genomic information and chemical and systems information was The Kyoto Encyclopedia of Genes and Genomes (KEGG). The GO and KEGG analyses were performed in the DAVID database [44,45,46,47,48], an integral functional annotation tool for revealing the biological significance behind a large list of genes, and a *p* value < 0.05 was set as the boundary criterion, as we previously described [49,50,51,52,53,54,55].

### 2.4. Survival Analysis

We determined correlations between LAGE gene family mRNA expression levels and the survival of BRCA patients as analyzed using the Kaplan Meier-plot database (https://kmplot.com, accessed on 30 November 2020). By focusing on distant metastasis, free survival (DMFS) clinical patients LAGE gene family expression with all default settings from the Kaplan Meier-Plot database [30], including survival curve, *p* log-rank value, and hazard ratios (HRs) with 95% confidence intervals (CIs), were all maintained on the plot.

### 2.5. Analysis of the Protein Expression in Clinical Human Specimens

LAGE family protein expressions were further evaluated using the publicly available Human Protein Atlas (HPA) platform, which contains images of tissue microarrays labeled with antibodies alongside 11,250 human proteins. These microarrays contain sections from 46 normal human tissues and more than 20 types of human cancer [56,57,58].

### 2.6. Statistical Analysis

In order to validate our analysis, we utilized TCGA Pan Cancer Atlas, a dataset from cBioportal (https://www.cbioportal.org, accessed on 30 November 2020), and extracted the clinical patient’s data to perform the effects of different LAGE family expression on overall survival analysis. A Cox proportional hazard model was carried out to evaluate the clinicopathological parameters through univariate and multivariate analysis. For survival analysis, the Kaplan-Meier plotter was applied, with all default settings, the Distant metastasis free survival (DMFS) was selected, with the Jetset as the best probe. Log-rank *p* value < 0.05 was considered to be statistically significant. The Tables S3–S6 in Supplementary described the parameters and its *p* value for MetaCore analysis, and the cut off *p* value < 0.01 was set.

## 3. Results

### 3.1. LAGE Family Expressions in Different Types of Cancer

**Figure 1:**
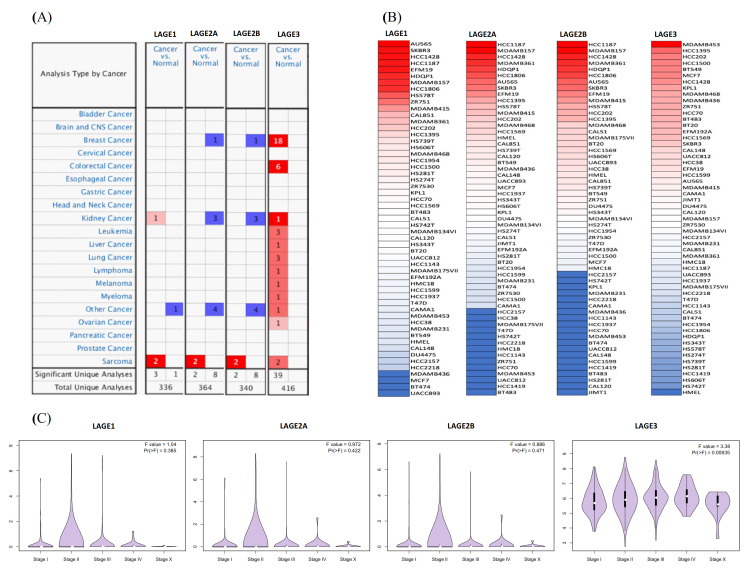
Expressions of L-antigen family genes through cancer tissues. (**A**) LAGE genes expression profiles of 20 types of cancer and normal patients. Decreasing gene rank percentiles are indicated as a color gradient. *p* value < 0.05, multiple of change of >1.5-fold, and a gene rank percentile of <10% in cancer compared to normal tissues were considered as threshold. (**B**) Heatmap plots collected from the CCLE database show LAGE expression levels in all breast cancer cell lines. Expression levels of LAGEs varied in different breast cancer cell lines. The up blocks with red color illustrate over-expression, and the down blocks showed under-expression. (**C**) Differential expressions of explicit LAGE family members were demonstrated in violin plots derived from the TCGA breast cancer database with a significant p-value set. By using TCGA and METABRIC databases, various stages of tumor across LAGE family gene expression profiles were explored. More elevated gene expression levels of LAGEs were found in higher-stage breast cancers than in lower-stage cancers and normal tissues. Only LAGE3 was clearly shown, whereas the others are still not clear.

We revealed mRNA expression from the LAGE gene family consisting of LAGE-1, LAGE2A, LAGE2B, and LAGE-3 in 20 cancer types (Figure 1). Among all these, the LAGE3 gene family was particularly overexpressed in breast cancer tissues compared to normal samples in 18 analyses (Figure 1A). Detail information of included datasets are displayed in Table S1 in Supplementary Materials. We then explored the expression levels of members of the LAGE gene family from the Cancer Cell Line Encyclopedia (CCLE) database. Based on CCLE analysis, triple-negative breast cancer cell lines, such as AU565, HCC1187 and MDAMB453 had high expression in LAGE-1, LAGE2A, LAGE2B and LAGE-3 (Figure 1B). LAGE3 expressions were confidently associated with metastatic events, tumor grades, and tumor stages in breast cancer patients with different stages (Figure 1C). To further explore the role of several clinicopathological factors in the development of breast cancer, we investigated the univariate and multivariate Cox hazard model. By collecting 1084 samples from the cBioportal platform and TCGA Pan Cancer Atlas dataset, we comprehensively evaluated the hazard model, clearly described in Table S2 and Figure S1 in Supplementary Materials.

**Figure 2:**
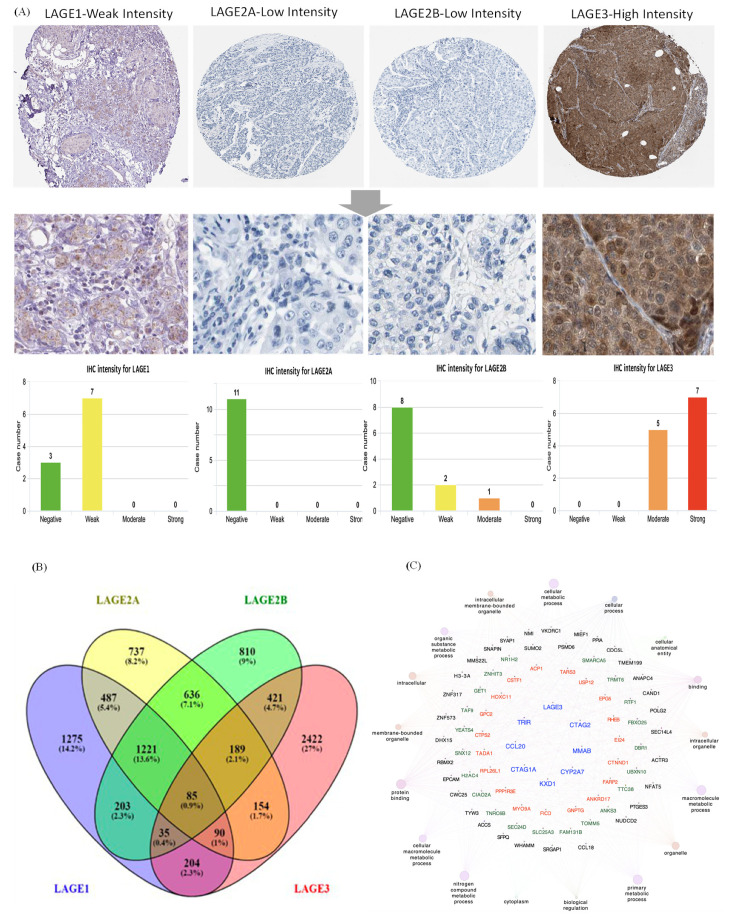
Expressions of L-antigen family genes throughout breast cancer tissues. (**A**) LAGE family genes, protein expressions in normal breast tissue, and breast cancer specimens from the Human Protein Atlas. Bar charts of the IHC staining intensity of LAGE family genes for breast cancer dictionary (10, 11, 11, 12 cases for LAGE1, LAGE2A, LAGE2B, LAGE3, respectively). (**B**) Venn diagram of LAGE family co-expression genes lists in TCGA breast cancer databases. The intersection of co-expression gene lists of each gene in the LAGE family. The approach was to select the top 20% of genes from the list of co-expressed genes extracted from the METABRIC dataset. (**C**) Through a Cytoscape analysis, thresholds were set to show pathways with *p*-value < 0.05, Network specificity as “Global”, Network Connectivity as “Medium”, and high correlations between LAGEs and metastasis markers were observed.

Since the expression of LAGE family genes was differentially expressed in breast cancer patients, we next performed further exploration of the potential roles of family genes in clinical human breast cancer specimens, connected with other featured biomarkers according to molecular subtypes of breast cancer. To determine the clinical relevance of LAGE family members’ expression, we analyzed the LAGE family members’ protein expression in clinical specimens from HPA. The data demonstrated that LAGE1 had weak expression and LAGE3 had the strong positive expression in breast cancer, and negative, weak expression in normal breast (Figure 2A). To determine the gene network’s co-expression between LAGE-1, LAGE2A, LAGE2B, and LAGE-3 in METABRIC data, the first step is to explore the LAGE genes’ co-expressed genes using Venny version 2.1.0 (https://bioinfogp.cnb.csic.es/tools/venny/, accessed on 30 November 2020). The results from the Venn diagram show 85 mutual genes beween LAGE members (Figure 2B). The 85 common genes were inputted to GlueGO and CluePedia to build a network of gene interactions and their related pathways. Through Cytoscape analysis (GlueGO and CluePedia), thresholds were set to show pathways with *p*-value < 0.05, Network specificity as “Global”, Network Connectivity as “Medium”, and we found that members of the LAGE gene family have a high correlation with metastatic markers such as FAM131B [59,60], CIAO2A [61], SNX12 [62,63] (Figure 2C).

### 3.2. LAGE-1 Is Overexpressed in Clinical Breast Cancer Datasets

**Figure 3:**
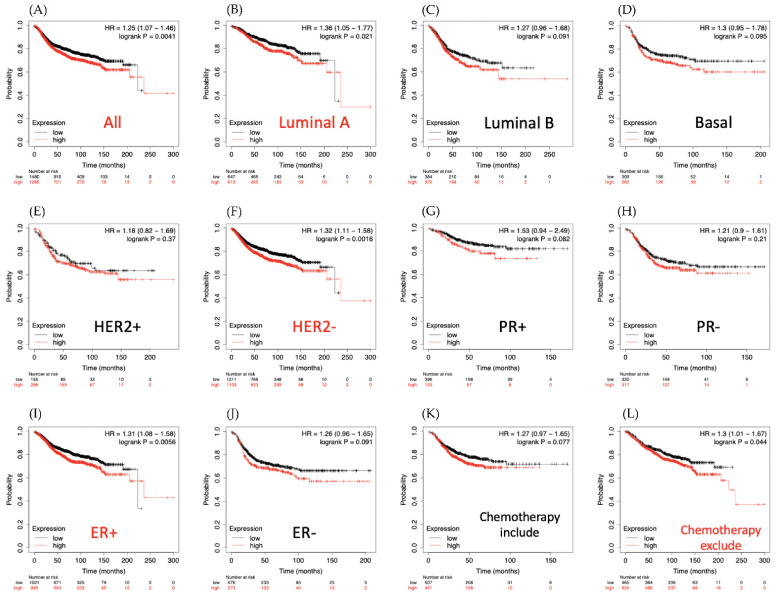
Distant metastasis-free survival (DMFS) analysis of L-antigen 1 (LAGE1) expression of clinical breast cancer cases. Kaplan-Meier plots, which are based on LAGE1 mRNA expression levels, displayed the DMFS prognosis of breast cancer patients. (**A**) Overall survival, (**B**) Luminal A subtype, (**C**) Luminal B subtype, (**D**) Basal subtype, (E) HER2^+^ subtype, (**F**) HER2^−^ subtype, (**G**) PR^+^ subtype, (**H**) PR^−^ subtype, (**I**) ER^+^ subtype, (**J**) ER^−^ subtype, (**K**) survival analysis for chemotherapy-treated patients and (**L**) chemotherapy untreated patients.An auto-cutoff was applied in this analysis to divide patients into two groups based on the best cutoff value of LAGE1 mRNA. Higher and lower expression levels of LAGE1 mRNA than the cutoff value are respectively shown in red and black. Significant correlations are shown between highly expressed LAGE1 and poor survival outcomes of breast cancer patients. The red titles of survival curves represent the significant *p* < 0.05, while the black titles were considered not statistically significant).

Associations of breast cancer survival with LAGE-1 expression are shown in Figure 3. Compared to those with low expression, patients with high expression of LAGE-1 had higher risk of shorter distant metastasis-free survival (DMFS) period in luminal A subtype (HR = 1.36, 95% CI: 1.05–1.77, *p* for trend = 0.021) (Figure 3B).

**Figure 4:**
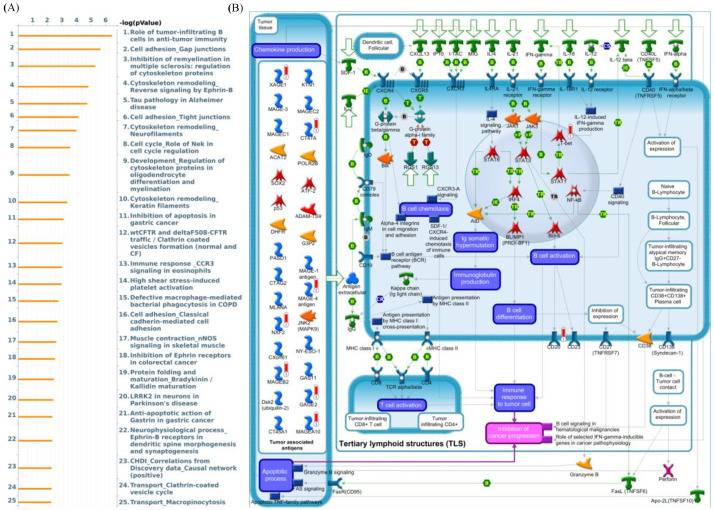
MetaCore enrichment pathway analysis of L-antigen 1 (LAGE1) co-expressed genes list in a breast cancer database. (**A**) In order to investigate potential gene networks and indicate pathways affected by the chosen genes, we exported LAGE1 co-expressed genes from TCGA and METABRIC breast cancer datasets to the MetaCore pathway analytical tool. (**B**) The “biology processes” analysis from MetaCore revealed that the “role of tumor-infiltrating B cells in the anti-tumor immunity-related pathway” was correlated with breast cancer development.

Moreover, these results also revealed that patients with chemotherapy treatment and overexpressed LAGE-1 had a higher survival probability than chemotherapy untreated patients (Figure 3K,L). In addition, GeneGo MetaCore’s explanation of the enriched biological processes showed that the genes expressed with LAGE-1 were associated with the cell cycle’s molecular processes. Furthermore, MetaCore has also become accustomed to build functional networks to specify the biological process involved in each tissue. After uploading the LAGE-1 co-expression genes list from the METABRIC and TCGA datasets into MetaCore program, we discovered that immune and adhesion-related pathway play critical roles in breast cancer development such as “Role of tumor-infiltrating B cells in anti-tumor immunity”, “Cell adhesion_Gap junctions”, “Inhibition of remyelination in multiple sclerosis: regulation of cytoskeleton proteins“, “Cell adhesion_Tight junctions“, “Immune response _CCR3 signaling in eosinophils” (Figure 4).

### 3.3. LAGE2A Expression Is Closely Involved in Poor Clinical Outcome

**Figure 5:**
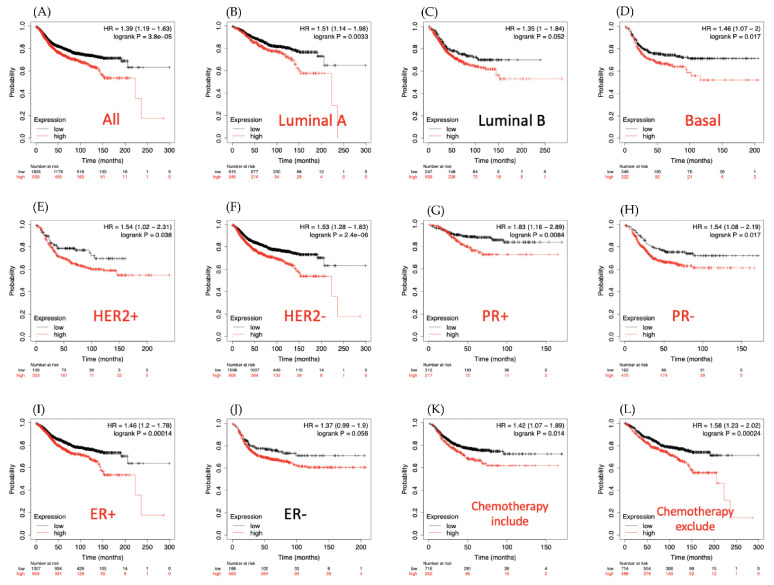
Survival analysis of LAGE2A expression over clinical breast cancer patients. Kaplan-Meier plots were used to indicate the distant metastasis-free survival (DMFS) prediction of breast cancer patients based on LAGE2A mRNA expression. (**A**) Overall survival, (**B**) Luminal A subtype, (**C**) Luminal B subtype, (**D**) Basal subtype, (**E**) HER2^+^ subtype, (**F**) HER2^−^ subtype, (**G**) PR^+^ subtype, (**H**) PR^−^ subtype, (**I**) ER^+^ subtype, (**J**) ER^−^ subtype, (**K**) survival analysis for chemotherapy-treated patients and (**L**) chemotherapy untreated patients. An auto-cutoff was applied in this analysis to divide patients into two groups based on the best cutoff value of LAGE2A mRNA. Higher and lower expression of LAGE2A mRNA than the cutoff value are respectively shown in red and black. Significant correlations (*p* < 0.05) are shown between highly expressed LAGE2A and poor survival outcomes of breast cancer patients. Similarly, significant curves were presented in red titles, and black titles showed non-significant results.

**Figure 6:**
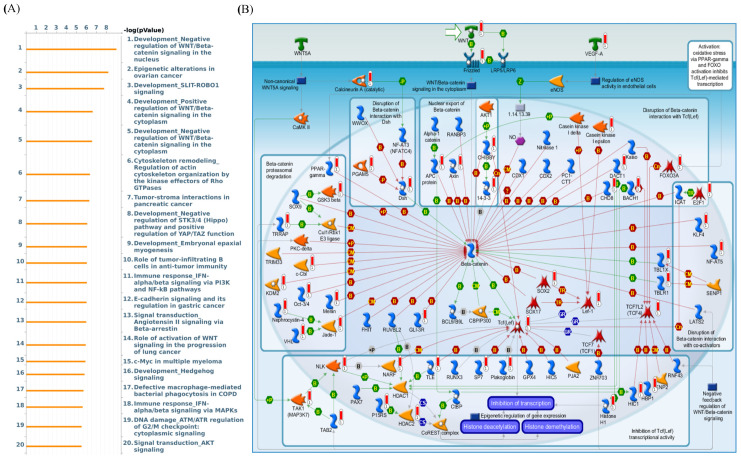
(**A**) In order to define the network of potential genes and related pathways affected by input genes, we exported LAGE2A co-expressed genes from TCGA and METABRIC breast cancer datasets to the MetaCore pathway analytical tool. (**B**) The result of MetaCore pathway analysis of “biology processes” illustrated that “development negative regulation of WNT/Beta catenin signaling” in the nucleus-related pathway was correlated with breast cancer development.

The Kaplan-Meier plot database also showed that for overall survival time, LAGE2A is overexpressed in DMFS of breast cancer patients and is associated with poor prognosis outcome (HR = 1.39, 95% CI: 1.19–1.63, *p* for trend = 3.8 × 10^−5^, Figure 5A). Similarly, in the basal, luminal A, HER2^+^, HER2^−^, PR^+^, ER^+^ survival curves indicated a worse survival time for LAGE2A high expression level with HR value were 1.46, 1.51, 1.54, 1.53, 1.83, 1.54, 1.46, respectively (Figure 5B,D–I). Among chemotheraphy excluded patients, high expression of LAGE2A has higher risk of shorter survival period when comparing to those had low LAGE2A expression. Next, MetaCore explanation of the enriched biological processes showed that the genes expressed with LAGE2A were involved in molecular processes related to the cell cycle. Again, we set the input of MetaCore by the LAGE2A co-expression gene from the METABRIC and TCGA database, and we observed that stemness related pathway play critical roles in breast cancer development such as “Development_Negative regulation of WNT/Beta-catenin signaling in the nucleus”, “Development_Negative regulation of STK3/4 (Hippo) pathway and positive regulation of YAP/TAZ function”, and “Development_Role of growth factors in the maintenance of embryonic stem cell pluripotency”. Meanwhile, LAGE2A also correlated with “Development_SLIT-ROBO1 signaling”, “Immune response_IFN-alpha/beta signaling via PI3K and NF-kB pathways”, “Signal transduction-Angiotensin II signaling via Beta-arrestin “, “Chemotaxis-Lysophosphatidic acid signaling via GPCRs”, and ”DNA damage_p53 activation by DNA damage” (Figure 6).

### 3.4. LAGE-2B Has a High Expression in Most Breast Cancer Subtypes

**Figure 7:**
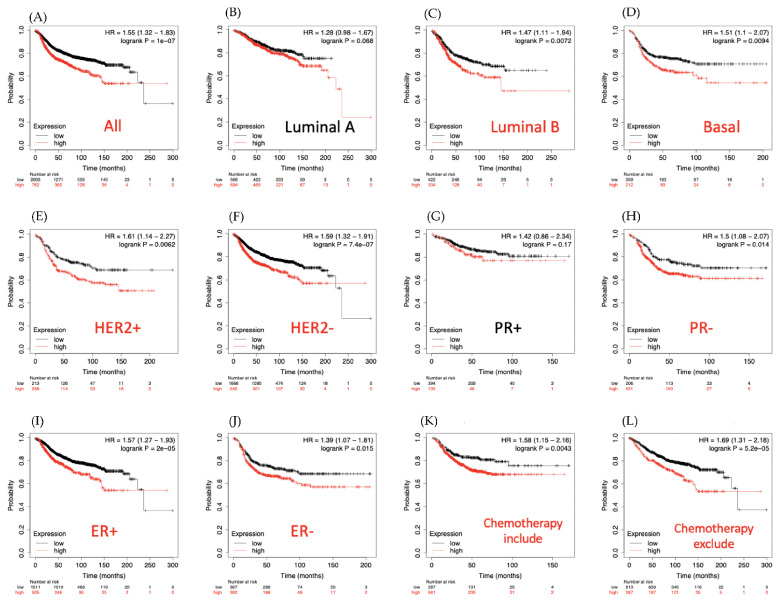
L-antigen 2B (LAGE2B) expression and distant metastasis-free survival (DMFS) of clinical breast cancer patients through survival analysis (*n* = 2898). Kaplan-Meier graphs were plotted based on LAGE2B mRNA expression levels and showing the DMFS prognoses of breast cancer patients. (**A**) Overall survival, (B) Luminal A subtype, (**C**) Luminal B subtype, (**D**) Basal subtype, (**E**) HER2^+^ subtype, (**F**) HER2^−^ subtype, (**G**) PR^+^ subtype, (H) PR^−^ subtype, (**I**) ER^+^ subtype, (**J**) ER^−^ subtype, (**K**) survival analysis for chemotherapy-treated patients and (**L**) chemotherapy untreated patients. For two groups of patients based on the best cutoff value of LAGE2B mRNA and coloring the groups red and black, an auto-cutoff function was applied. High expression of LAGE2B was highly correlated with poor survival outcomes of breast cancer patients (*p* < 0.05 was considered statistically significant).

**Figure 8:**
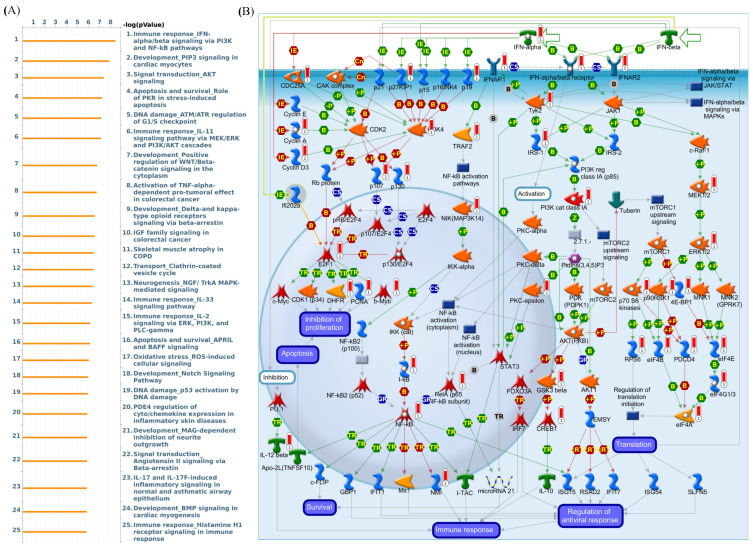
L-Antigen 2B (LAGE2B) is particularly overexpressed in breast cancer patients. (**A**) In order to discover prospective gene networks and pathways impacted by the chosen genes, we exported LAGE2B co-expressed genes from TCGA and METABRIC breast cancer datasets to the MetaCore pathway analytical tool. (**B**) The MetaCore pathway analysis of “biology processes” indicated that “immune response_IFN-alpha/beta signaling via PI3K and NF-κB”-related pathways were correlated with breast cancer development.

The Kaplan-Meier tool also showed that in DMFS breast cancer patients, overexpressed level of LAGE2B led to poor prognosis in overall survival (HR = 1.55, 95% CI: 1.32–1.83, *p* for trend = 1 × 10^−7^, Figure 7A). Results in the basal (HR = 1.51 (1.1–2.07), *p* = 0.0094), luminal subtype B (HR = 1.47 (1.11–1.94), *p* = 0.0072), HER^+^ (HR = 1.61 (1.11–2.27), *p* = 0.0062), HER^−^ (HR = 1.59 (1.32–1.91), *p* = 7.4 × 10^−7^), PR^−^ (HR = 1.5 (1.08–2.07), *p* = 0.014), ER^+^ (HR = 1.57 (1.27–1.93), *p* = 1 × 10^−5^), ER^−^ (HR = 1.39 (1.07–1.81), *p* = 0.015) also described the worse survival rate for LAGE2B overexpressed patients. For chemotherapy-treated patients and untreated patients, there was no difference in survival time, and both had poor outcomes for overexpression of LAGE2B. Next, MetaCore explanation of the enriched biological processes revealed that the genes expressed with LAGE2B were involved in molecular processes related to the cell cycle. Furthermore, MetaCore has become familiarized with making pathways networked from the input genes list to stipulate biological processes. Selecting the LAGE2A co-expression gene from the METABRIC and TCGA database into MetaCore platform, we found that the MAPK and Apoptosis related pathway play critical roles in breast cancer development such as “Immune response IFN-alpha/beta signaling via PI3K and NF-kB pathways”, “Development-PIP3 signaling in cardiac myocytes”, “Signal transduction-AKT signaling”, “Apoptosis and survival-Role of PKR in stress-induced apoptosis”, “DNA damage_ATM/ATR regulation of G1/S checkpoint”, “Immune response-IL-11 signaling pathway via MEK/ERK and PI3K/AKT cascades”, “Development-Positive regulation of WNT/Beta-catenin signaling in the cytoplasm”, “Activation of TNF-alpha-dependent pro-tumoral effect in colorectal cancer”, “Development-Delta- and kappa-type opioid receptors signaling via beta-arrestin”, “IGF family signaling in colorectal cancer”, “Skeletal muscle atrophy in COPD”, “Transport-Clathrin-coated vesicle cycle”, “Neurogenesis NGF/TrkA MAPK-mediated signaling”, “Immune response IL-33 signaling pathway”, and “Immune response IL-2 signaling via ERK, PI3K, and PLC-gamma” (Figure 8).

### 3.5. LAGE3 Is Significantly Overexpressed in Breast Cancer Patients

**Figure 9:**
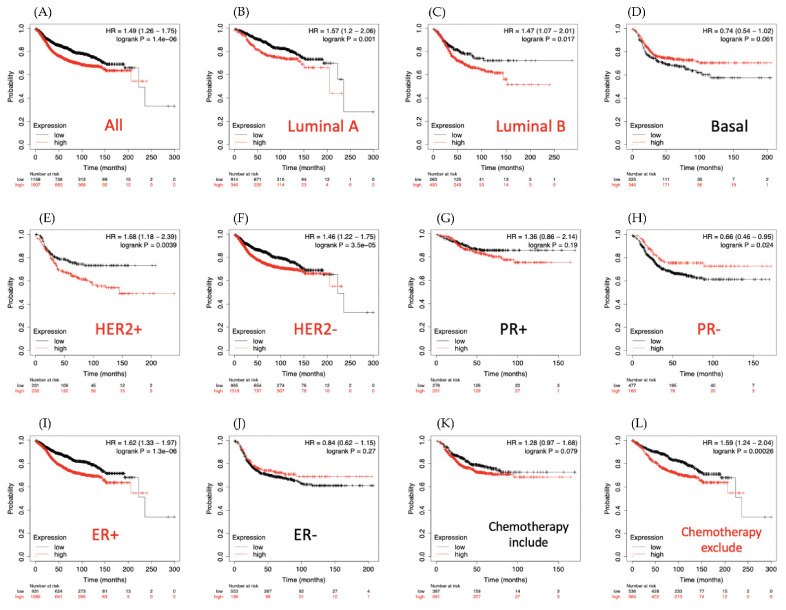
Relationship of distant metastasis-free survival (DMFS) and LAGE3 expression levels with clinical breast cancer patients (*n* = 2898). DMFS prognoses of clinical BRCA patients are shown in Kaplan-Meier graphs, based on LAGE3 mRNA expression levels. (**A**) Overall survival, (**B**) Luminal A subtype, (**C**) Luminal B subtype, (**D**) Basal subtype, (**E**) HER2^+^ subtype, (**F**) HER2^−^ subtype, (**G**) PR^+^ subtype, (**H**) PR^−^ subtype, (**I**) ER^+^ subtype, (**J**) ER^−^ subtype, (**K**) survival analysis for chemotherapy-treated patients and (**L**) chemotherapy untreated patients. An auto-cutoff applied in this analysis helped to create two groups of patients based on the best cutoff value of LAGE3 mRNA, as demonstrated in red (high) and black (low). LAGE3 with high expression levels was considerably correlated with poor survival outcomes; the red label of survival curves stands for significant *p*-value, whereas black label indicates a non-significant outcome.

**Figure 10:**
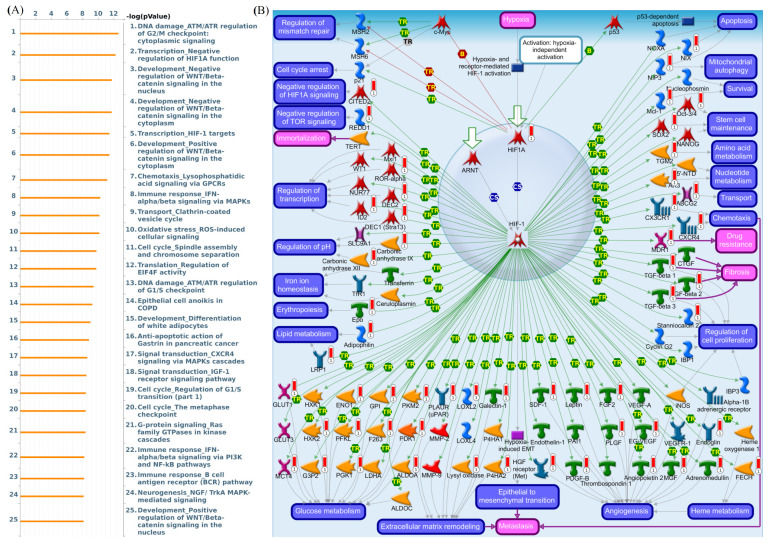
MetaCore pathway analysis of L-antigen 3 (LAGE3) co-expressed genes in a breast cancer database. (**A**) We exported LAGE3 co-expressed genes from TCGA and METABRIC breast cancer datasets to the MetaCore pathway analytical tool to find the relationship between our selected genes and enriched biological pathways. (**B**) The MetaCore pathway analysis of “biology processes” and “transcription_HIF-1 targets”-related pathways were correlated with breast cancer development.

As shown in survival plots in Figure 9, compared with low LAGE3 expression group, there was a lower rate of overall survival in the high expression group of patients (HR = 1.49, 95% CI: 1.26–1.75, *p* for trend = 1.4 × 10^−6^, Figure 9A). Similarly, in luminal A (HR = 1.57 (1.2–2.06), *p* = 0.001), luminal B (HR = 1.47 (1.07–2.01), *p* = 0.017), HER2^+^ (HR = 1.68 (1.18–2.39), *p* = 0.0039), HER2^−^ (HR = 1.46 (1.22–1.75), *p* = 3.5 × 10^−5^), and ER^+^ (HR = 1.62 (1.33–1.97), *p* = 1.3 × 10^−6^) survival curves indicated a worse survival time for high expression of LAGE3 in patients. Interestingly, high LAGE3 expression was associated with poor survival outcomes for the group of chemotherapy untreated patients. Thus, MetaCore explanation of the enriched biological processes showed that the genes expressed with LAGE3 were involved in molecular processes related to the cell cycle. We added LAGE3 co-expression genes list from the TCGA and METABRIC database into MetaCore software, then observed that DNA damage and HIF-1 related pathway play critical roles in breast cancer development such as “DNA damage_ATM/ATR regulation of G2/M checkpoint: cytoplasmic signaling”, “Transcription_Negative regulation of HIF1A function”, “Development_Positive regulation of WNT/Beta-catenin signaling in the cytoplasm”, “Chemotaxis_Lysophosphatidic acid signaling via GPCRs”, “Immune response_IFN-alpha/beta signaling via MAPKs”, “Transport_Clathrin-coated vesicle cycle”, “Oxidative stress_ROS-induced cellular signaling”, “Translation_Regulation of EIF4F activity”, “DNA damage_ATM/ATR regulation of G1/S checkpoint”, and “Epithelial cell anoikis in COPD” (Figure 10).

## 4. Discussion

BRCA has the highest prevalence compared to most other types of cancer, particularly in women [64,65]. Despite years of extraordinary efforts to enhance our knowledge of its biology and improving surgical treatments, and chemotherapies, patient prognoses with advanced BRCA have not yet been clarified [66]. Therefore, it is pivotal to determine new biomarkers to improve patient prognoses and develop effectual interpositions. In the present study, we focused on the LAGE family, which is expressed in many human organs and cell types. LAGE family members are crucial in RNA polymerase II-mediated positive transcription regulation and transfer (t)RNA threonyl-carbamoyl adenosine metabolic processes [17]. Overexpression of LAGE family mRNAs was also found in sarcomas, lung cancer, colorectal cancer, and kidney cancer. Our study aimed to systematically dissect the biological functions and associated regulatory pathways of the LAGE family in BRCA by employing a more-comprehensive analysis from open-access databases. To develop practical therapeutic approaches and novel prognostic strategies, it is necessary but challenging to illustrate the pathogenesis of BRCA and decipher the occurrence of transformation from normal to cancer cells. Since some of the LAGE family members present high expression levels in different cancer types, this family was proposed to be associated with tumor progression and thus may be a potential therapeutic target [67]. To the best of our knowledge, this is the first study to inspect associations of LAGE family members exhibiting distinct gene expression levels with adverse clinicopathological factors and prognoses of BRCA. Our results suggested that LAGEs have the potential to serve as prognosticators in BRCA.

We conducted co-expression and functional enrichment analyses in order to demonstrate the biological functions of LAGE gene family members and their associated regulatory pathways via setting as input LAGE co-expressing genes from the METABRIC and TCGA databases into the Metacore platform. Previous analyses of published databases showed that 25%–50% of LAGE1 samples were expressed in melanoma tumors, non-small cell lung carcinomas, and head, bladder, neck, and prostate cancers, and so LAGE1 mRNA expression was found in 22 of 107 (21%) tumor tissues [68,69]. Our results revealed a correlation between immune and adhesion-related pathways with LAGE1 in BRCA. Correlations between higher tumor-infiltrating B cell densities and improved clinical outcomes were discussed in a previous study [70]. Many immune cell populations create a complex environment for tumor development, and the capacity of the immune system to detect the tumor cells has been reflected [71]. The characterization of this tumor microenvironment can help to describe tertiary lymphoid structures (TLS). TLS characterize sites of lymphoid neogenesis that are acquired in almost all solid cancers such as breast cancer, non-small cell lung cancer, colorectal cancer, pancreatic and gastric carcinoma [71,72]. Besides, research has suggested that LAGE1 (also known as CTAG2) is necessary for directional migration, and has the essential function of promoting collective invasion, primary tumor growth and pulmonary metastasis [73].

We observed that through MetaCore analysis, LAGE2A and its several pathways were significantly associated with breast cancer development. For example, the canonical WNT/Beta-catenin signaling pathway plays instructive roles in animal growth. The duration or intensity of a WNT-initiated signal can be limited by negative feedback loops. These responses are induced via G-proteins, WNT-activated kinases, such as Tcf(Lef) mediated transcription of negative regulators, Casein kinase II and some other mechanisms. Some studies found that LAGE2A has a high expression at protein level, as well as playing a crucial role in oncogenesis such as in resected lung cancer [17] and Esophageal Squamous Cell Carcinoma [74]. They also discovered the unique role of cancer-testis antigen in targeting cancer stem cells [75], and for further cancer treatment [12]. Consistently, our findings underline the proof that this family of genes could be an effective biomarker for breast cancer patient treatment.

Furthermore, we found that MAPK- and apoptosis-related pathways played critical roles in BRCA development such as “immune response_IFN-alpha/beta signaling via PI3K and NF-κB pathways”. Type I interferons (IFNs) have several important biological activities such as antiviral, antiproliferative and pro-apoptotic functions. The antiproliferative role is mainly via increased expression of cyclin-dependent kinase inhibitors such as p21, and increased activity of Rb protein, which is an inhibitor of the cell cycle progression transcription factor E2F1 [76]. NF-κB signaling pathway partially protects against IFNs induced apoptosis, plays significant roles in the development and progression of BRCA and could perform as potential target for BRCA prevention and treatment [77]. Consistently, several studies have described LAGE2B as an immunogenic cancer-testis gene relevant to spontaneous and vaccine-induced immunity, correlated to tumor proliferation [78,79].

Interestingly, LAGE3 showed a strong signature to be a potential biomarker for breast cancer in the results of our study. First of all, this gene is upregulated in 18 analyses of breast cancer patients, which met our thresholds from 6 datasets. Our method provides an observation of correlations between LAGE3 expression and BRCA malignancy, and revealed that high LAGE3 expression was significantly related to different stages of BRCA. Indeed, previous studies demonstrated LAGE3 expression in other tumor cells [15,16], which is consistent with our findings. Secondly, the human protein atlas also revealed that LAGE3 has a higher proportion of moderate and strong intensity in breast cancer samples relative to normal breast tissues. We also found that DNA damage and HIF-1-related pathways play critical roles in BRCA development via LAGE3 co-expressing genes. A high G_2_/M pathway score was shown to be correlated with clinically destructive features of tumors and patient survival in a previous study [80]. The roles of HIF-1 in regulating BRCA cell metastasis, its effects on metastasis, and therapeutic opportunities for BRCA were discussed elsewhere [81].

In addition, multivariate analysis indicated that “age” and “tumor stage” were significantly associated with high-risk factors, and LAGE3 expression levels are an independent survival determinant in patients with BRCA (Supplementary Figure S1). Thus, our results suggested that LAGE3 is upregulated in many BRCA cases, and can potentially serve as a diagnostic and prognostic marker; further clinical validation of this finding is still required. Moreover, through computational analysis, expression patterns of genes in cancer and normal tissues were explicitly evaluated to help us further establish the role of LAGE3 in breast cancer tissue growth. Therefore, LAGE3 could be a prognosticative biomarker and curative target in BRCA.

## 5. Conclusions

In summary, although many observations have demonstrated that LAGE1, LAGE2A, LAGE2B play crucial roles in breast cancer maturity, this still needs further exploration to verify effectiveness in breast cancer patients. By using a meta-analysis approach, our study suggested that, in LAGE family genes, LAGE3 has a prospective value and maybe a new biomarker and therapeutic target for future breast cancer treatment.

## Data Availability

Oncomine: http://oncomine.org; cBioportal: https://cbioportal.org; The Human Protein Atlas: https://www.proteinatlas.org; Kaplan Meier-plot database https://kmplot.com, MetaCore Analysis https:/portal.genego.com The datasets used and/or analyzed during the current study (accessed on 30 November 2020), which are available from the corresponding author on reasonable request.

## Supplementary Materials

The following are available online at https://www.mdpi.com/article/10.3390/diagnostics11040726/s1, Figure S1: Multivariate analysis of LAGE3 expression and the relationship between it and clinicopathological parameters (Age, Treatment, Stage, TNM stage). Table S1: Basic characteristic of LAGE3 gene on Oncomine database. Table S2: Univariate of LAGE3 expression and its clinicopathological parameters. Table S3: Pathway analysis of LAGE1-coexpressed genes from public breast cancer databases using the MetaCore database (*p* < 0.01 set as the cutoff value). Table S4: Pathway analysis of LAGE2A-coexpressed genes from public breast cancer databases using the MetaCore database (*p* < 0.01 set as the cutoff value). Table S5. Pathway analysis of LAGE2B-coexpressed genes from public breast cancer databases using the MetaCore database (*p* < 0.01 set as the cutoff value). Table S6. Pathway analysis of LAGE3-coexpressed genes from public breast cancer databases using the MetaCore database (*p* < 0.01 set as the cutoff value).
