# Supplementary material for: Analysis of LAGEs Family Gene Signature and Prognostic Relevance in Breast Cancer
Source: Diagnostics (Basel). 2021 Apr 19;11(4):726. doi: 10.3390/diagnostics11040726 (PMC8074247; doi:10.3390/diagnostics11040726)
Supplement: Supplementary file 1 [file diagnostics-11-00726-s001.zip › diagnostics-1183200-Supplementary data.pdf]

Supplementary data:

Table S1. Basic characteristic of LAGE3 gene on Oncomine database

Gene	*Dataset*	*group comparison*	*fold change*	*p-value*	*number of patients*
**LAGE3**	Curtis breast (n=2136)	Invasive Lobular Breast Carcinoma vs. Normal	2.986	3.89E-72	148/292
		Invasive Ductal and Invasive Lobular Breast Carcinoma vs. Normal	2.984	7.40E-39	90/234
		Invasive Breast Carcinoma vs. Normal	2.516	5.24E-09	21/165
		Tubular Breast Carcinoma vs. Normal	2.844	1.17E-34	67/211
		Invasive Ductal Breast Carcinoma vs. Normal	3.042	9.69E-102	1556/1700
		Breast Carcinoma vs. Normal	2.648	2.91E-06	14/158
		Medullary Breast Carcinoma vs. Normal	2.803	2.64E-12	32/176
		Ductal Breast Carcinoma in Situ vs. Normal	2.851	8.94E-05	10/154
	TCGA breast (n=593)	Invasive Ductal Breast Carcinoma vs. Normal	2.686	1.91E-69	389/450
		Invasive Breast Carcinoma vs. Normal	2.387	1.22E-34	76/137
		Invasive Lobular Breast Carcinoma vs. Normal	2.648	5.03E-17	36/97
		Male Breast Carcinoma vs. Normal	4.033	1.99E-08	3/64
		Mixed Lobular and Ductal Breast Carcinoma vs. Normal	2.504	9.74E-05	7/68
	Zhao Breast (n=64)	Lobular Breast Carcinoma vs. Normal	3.01	2.04E-09	21/24
		Invasive Ductal Breast Carcinoma vs. Normal	3.073	1.12E-10	38/41
	Perou Breast (n=65)	Ductal Breast Carcinoma vs. Normal	2.313	1.15E-07	36/39
	Richardson Breast 2 (n=47)	Ductal Breast Carcinoma vs. Normal	3.254	1.00E-11	40/47
	Sorlie breast (n=85)	Lobular Breast Carcinoma vs. Normal	2.734	5.62E-05	65/69

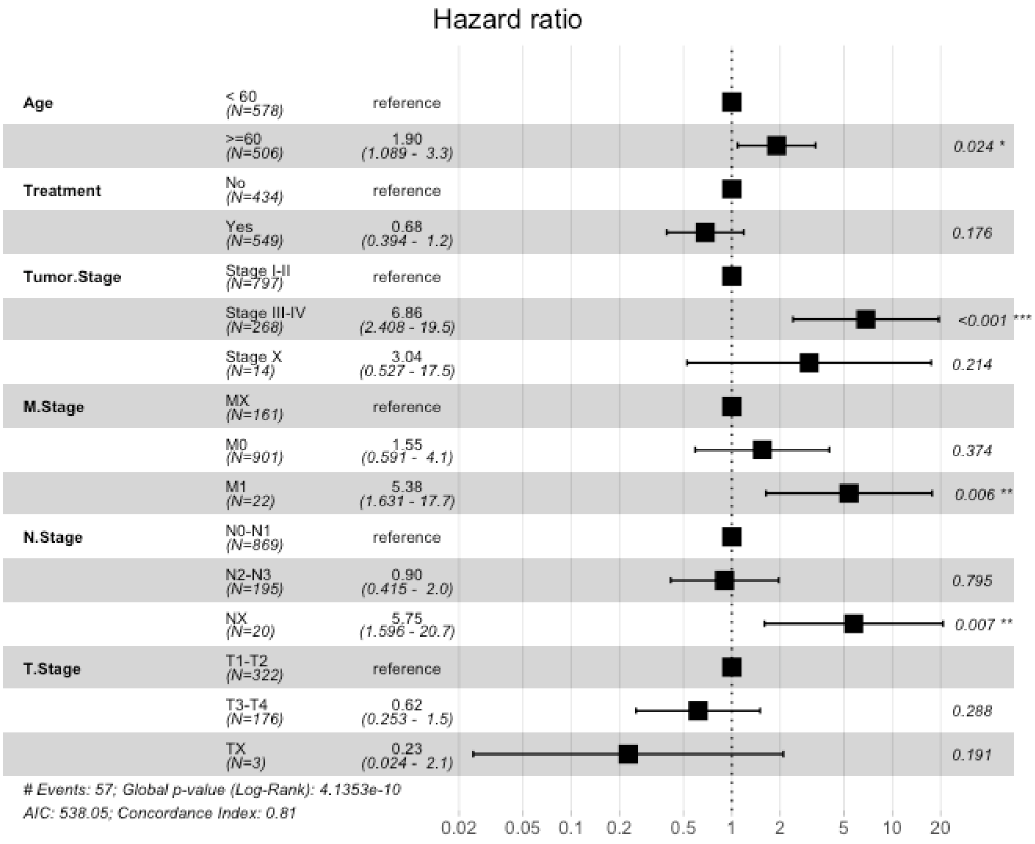

Figure S1. Multivariate analysis of LAGE3 expression and the relationship between it and clinicopathological parameters (Age, Treatment, Stage, TNM stage)

Table S2. Univariate of LAGE3 expression and its clinicopathological parameters

element		number of samples	P-value	Hazard ratio	95% CI low	95% CI high
Age	<60	578				
	>=60	506	3.84E-05	1.97	1.427	2.721
Gender	Female	1072				
	Male	12	0.85	0.83	0.1155	5.927
Treatment	No	434				
	Yes	549	0.0352	0.57	0.3915	0.8318
Stage	Stage I-II	797				
	Stage III-IV	268	6.87E-09	2.671	1.916	3.723
	Stage X	14	0.0106	2.777	1.269	0.076
M Stage	MX	161				
	M0	901	0.73	0.9064	0.5188	1.584
	M1	22	4.27E-05	4.5076	2.1916	9.271
N Stage	N0-N1	869				
	N2-N3	195	1.34E-05	2.32	1.588	3.39
	NX	20	1.10E-05	4.06	2.174	7.583
T Stage	T1-T2	322				
	T3-T4	176	3.81E-03	1.9385	1.23814	3.035
	TX	3	6.81E-01	0.6568	0.08868	4.865

Table S3. Pathway analysis of LAGE1-coexpressed genes from public breast cancer databases using the MetaCore database ($p<0.01$ set as the cutoff value)

#	Maps	p-value	Network Objects from Active Data
1	Role of tumor-infiltrating B cells in anti-tumor immunity	3.73E-07	MAGEB2, MAGE-4 antigen, XAGE1, MAGEA10, NXF2, CT47A, CD20, GAGE2, T-bet
2	Cell adhesion_Gap junctions	2.43E-06	Tubulin beta, Tubulin alpha, Actin cytoskeletal, Tubulin (in microtubules), Actin
3	Inhibition of remyelination in multiple sclerosis: regulation of cytoskeleton proteins	5.38E-06	Tubulin beta, MARCKS, Tubulin alpha, p190RhoGAP, Actin cytoskeletal, Tubulin (in microtubules)
4	Cytoskeleton remodeling_Reverse signaling by Ephrin-B	1.71E-05	Ephrin-B receptors, Tubulin alpha, Actin cytoskeletal, F-Actin, Tubulin (in microtubules)
5	Tau pathology in Alzheimer disease	2.02E-05	p38 MAPK, Caspase-6, p38gamma (MAPK12), PP2C, DYRK1a, Tubulin (in microtubules)

6	Cell adhesion_Tight junctions	8.37E-05	Tubulin alpha, Actin cytoskeletal, F-Actin, Tubulin (in microtubules), Actin
7	Cytoskeleton remodeling_Neurofilaments	0.000114	Tubulin beta, Tubulin alpha, Actin cytoskeletal, Tubulin (in microtubules)
8	Cell cycle_Role of Nek in cell cycle regulation	0.000307	Insulin receptor, Tubulin beta, Tubulin alpha, Tubulin (in microtubules)
9	Development_Regulation of cytoskeleton proteins in oligodendrocyte differentiation and myelination	0.000341	Tubulin beta, Tubulin alpha, p190RhoGAP, Actin cytoskeletal, Tubulin (in microtubules)
10	Cytoskeleton remodeling_Keratin filaments	0.000487	Tubulin beta, Tubulin alpha, Actin cytoskeletal, Tubulin (in microtubules)
11	Inhibition of apoptosis in gastric cancer	0.000882	Gastrin 17, Gastrin 17-Gly, HGF receptor (Met), Progastrin
12	wtCFTR and deltaF508-CFTR traffic / Clathrin coated vesicles formation (normal and CF)	0.001065	Myosin I, Actin cytoskeletal, Actin
13	Immune response _CCR3 signaling in eosinophils	0.001162	p38 MAPK, Profilin I, Profilin, FGR, Actin cytoskeletal
14	High shear stress-induced platelet activation	0.001246	GP-IB alpha, Tyro3, Ephrin-B receptor 1, Actin cytoskeletal
15	Defective macrophage-mediated bacterial phagocytosis in COPD	0.00207	Tubulin alpha, MANR, Tubulin (in microtubules)
16	Cell adhesion_Classical cadherin-mediated cell adhesion	0.002322	F-Actin cytoskeleton, Actin cytoskeletal, F-Actin
17	Muscle contraction_nNOS signaling in skeletal muscle	0.002883	Syntrophin A, CACNA1C, Actin
18	Inhibition of Ephrin receptors in colorectal cancer	0.003519	Ephrin-B receptors, Ephrin-B receptor 2, Ephrin-B receptor 1
19	Protein folding and maturation_Bradykinin / Kallidin maturation	0.004236	Aminopeptidase P2, Tissue kallikreins, CPB2
20	LRRK2 in neurons in Parkinson's disease	0.004625	ACTB, Actin cytoskeletal, Tubulin (in microtubules)
21	Anti-apoptotic action of Gastrin in gastric cancer	0.005466	Gastrin 17, Gastrin 17-Gly, Progastrin
22	Neurophysiological process_Ephrin-B receptors in dendritic spine morphogenesis and synaptogenesis	0.005466	Ephrin-B receptors, Ephrin-B receptor 2, Actin cytoskeletal
23	CHDI_Correlations from Discovery data_Causal network (positive)	0.005918	Ephrin-B receptors, Actin cytoskeletal, Actin
24	Transport_Clathrin-coated vesicle cycle	0.006092	Myosin I, Actin cytoskeletal, VAMP8, Actin
25	Transport_Macropinocytosis	0.006472	Actin cytoskeletal, Tubulin (in microtubules)
26	Development_SLIT-ROBO1 signaling	0.00795	F-Actin cytoskeleton, ACTB, p190RhoGAP
27	Cytoskeleton remodeling_Hyaluronic acid/ CD44 signaling pathways	0.009711	HGF receptor (Met), Actin cytoskeletal, Actin
28	Role of platelets in allograft rejection	0.009711	GP-IB alpha, Glycoprotein VI, Kainate receptor

29	Proliferative action of Gastrin in pancreatic cancer	0.010344	Gastrin 17, Gastrin 17-Gly, Progastrin
30	Cytoskeleton remodeling_Substance P mediated membrane blebbing	0.011455	Tubulin alpha, Tubulin (in microtubules)
31	Neurophysiological process_Receptor-mediated axon growth repulsion	0.011682	F-Actin cytoskeleton, Actin cytoskeletal, Tubulin (in microtubules)
32	Signal transduction_Intracellular calcium increase	0.011682	CACNA1C, RAP-2B, PLC-delta 1
33	Cytoskeleton remodeling_Regulation of actin cytoskeleton nucleation and polymerization by Rho GTPases	0.011682	F-Actin cytoskeleton, Profilin, Actin cytoskeletal
34	Chemotaxis_Lipoxin inhibitory action on Formyl-Met-Leu-Phe-induced neutrophil chemotaxis	0.012386	p38 MAPK, F-Actin cytoskeleton, Actin cytoskeletal
35	Immune response_Fc gamma R-mediated phagocytosis in macrophages	0.012386	F-Actin cytoskeleton, Profilin, Actin cytoskeletal
36	Transcription_Transcription factor Tubby signaling pathways	0.012896	Insulin receptor, HTR2C (HTR1C)
37	Involvement of VEGF signaling in the progression of lung cancer	0.012896	p38 MAPK, p190RhoGAP
38	PI3K signaling in gastric cancer	0.014643	Gastrin 17, HGF receptor (Met), Progastrin
39	DNA damage_ATM/ATR regulation of G2/M checkpoint: cytoplasmic signaling	0.015444	p38 MAPK, p38gamma (MAPK12), PLK1
40	Proliferative action of Gastrin in gastric cancer	0.017118	Gastrin 17, Gastrin 17-Gly, Progastrin
41	Immune response_CCL2 signaling	0.017991	CCBP2 (CCR9), Actin cytoskeletal, CDH19
42	Cell cycle_Chromosome condensation in prometaphase	0.019388	BRRN1, CAP-H/H2
43	Platelet activation as a result of endothelial dysfunction after stenting	0.019812	Prostacyclin receptor, Glycoprotein VI, Actin cytoskeletal
44	CCR7 signaling pathways in dendritic cells in allergic contact dermatitis	0.020759	p38 MAPK, Profilin, Actin cytoskeletal
45	Development_H3K9 demethylases in pluripotency maintenance of stem cells	0.021185	Oct-3/4, ZFP57
46	NMDA-independent presynaptic long-term potentiation in Huntington's disease	0.021731	MARCKS, GluR7, Actin cytoskeletal
47	Cytoskeleton remodeling_CDC42 in cellular processes	0.023048	F-Actin cytoskeleton, Actin cytoskeletal
48	Oxidative stress_ROS-mediated MAPK activation via canonical pathways	0.023747	p38 MAPK, TRPM2, SFK
49	Stimulation of gastric acid secretion in gastric cancer	0.023747	Gastrin 17, Gastrin 17-Gly, Progastrin

50	Immune response_Fc epsilon RI pathway: signaling through Fyn and PI3K	0.024793	Fc epsilon RI beta, FGR, VAMP8

Table S4. Pathway analysis of LAGE2A-coexpressed genes from public breast cancer databases using the MetaCore database (p<0.01 set as the cutoff value)

#	Maps	pValue	Network Objects from Active Data
1	Development_Negative regulation of WNT/Beta-catenin signaling in the nucleus	1.02E-09	TBL1X, SP7, Calcineurin A (catalytic), BACH1, HBP1, Oct-3/4, VHL, APC protein, Lef-1, PGAM5, TCF7L2 (TCF4), 14-3-3, Jade-1, Casein kinase I epsilon, VEGF-A, TLE, Dsh, KLF4, Menin, NF-AT5, GSK3 beta, Nephrocystin-4, HDAC2, HIC1, P15RS, TBLR1, TAB2, PPAR-gamma, Axin, TAK1(MAP3K7), KDM2, TRRAP, NARF, c-Cbl, Tcf(Lef), E2F1, GLI-3R, CHD8, NLK, SOX2, WNT, FOXO3A, CHIBBY, Plakoglobin, Frizzled, Histone H1, DACT1
2	Epigenetic alterations in ovarian cancer	6.92E-09	DNMT3B, RARbeta, ZIC4, APC protein, CDC20, GATA-4, HDAC3, p21, DAB2, ESR2 (nuclear), CTCF, WIF1, FGFR1, STAR, Bax, OBCAM, HDAC2, RASSF1, CARD5, IGF-2, AL1A1, SKP2, HSD3B2, p27KIP1, Histone H3, GATA-6, DAPK1, SF1, ErbB4, LSD1, DOK2, ZIC1, Dlec1, Claudin-4, SSTR1, SUZ12, p15, GATA-5, Aurora-B, SNAIL1, L1TD1, GLI-1, MLH1
3	Development_SLIT-ROBO1 signaling	1.83E-08	Rictor, Calcineurin A (catalytic), NCK1, VEGFR-2, LSP1, PI3K reg class IA (p85), MENA, CDC42, Fyn, AKT(PKB), Cytohesin1, CXCR4, ACTB, RhoA, SRGAP1, F-Actin cytoskeleton, p190RhoGAP, SLIT3, Myosin II, ROCK, Calmodulin, FLII, SLIT1, SSH1L, Cofilin, PREX1
4	Development_Positive regulation of WNT/Beta-catenin signaling in the cytoplasm	2.63E-07	Casein kinase II, alpha chains, TBL1X, UBE2B, PP1-cat, APC protein, 14-3-3, TGIF, Makorin-1, Dsh, ZBED3, Beta-arrestin2, YAP1 (YAp65), PPP2R2A, SIAH1, USP25, Insulin receptor, GSK3 alpha/beta, TBLR1, AKT(PKB), RNF146, Axin, Tcf(Lef), 14-3-3 zeta/delta, PP2A catalytic, RNF220, ILK, DOCK4, Trabid, Tankyrases, WNT, USP7, FAK1, JNK(MAPK8-10), Parathyroid hormone, MITF, PKA-cat (cAMP-dependent), Frizzled, DACT1
5	Development_Negative regulation of WNT/Beta-catenin signaling in the cytoplasm	3E-07	CXXC4, VHL, PP1-cat, APC protein, Presenilin 1, Laforin, FAF1, Casein kinase I epsilon, CYLD, PI3K cat class III (Vps34), DAB2, Dsh, YAP1 (YAp65), G-protein alpha-13, LATS1, STK4, SIAH1, Itch, GSK3 alpha/beta, Ankyrin-G, WDR26, Skp2/TrCP/FBXW, TAZ, Axin, Prickle-1, c-Cbl, Tcf(Lef), E2F1, Amer1, PP2A catalytic, JNK1(MAPK8), RNF185, YAP1/TAZ, ELAVL1 (HuR), PEG3, Malin, WNT, NEDD4L, Frizzled, DACT1
6	Cytoskeleton remodeling_Regulation of actin cytoskeleton	5E-07	Spectrin, PRK1, Alpha-actinin, Talin, MLCP (reg), GIT1, Cdc42 subfamily, ERM proteins, RhoA-related, CDC42, Actin cytoskeletal, MLCK, BETA-PIX, RhoA, Citron,

	organization by the kinase effectors of Rho GTPases		Destrin, F-Actin cytoskeleton, Myosin II, RhoB, MyHC, Rac3, MRCK, ROCK, Actomyosin, PAK, Rac1-related, Rhov, MRLC, Filamin A, TC10, Cofilin
7	Tumor-stroma interactions in pancreatic cancer	5.81E-07	Galectin-3, PDGF-B, VEGF-A, KIF27, Fibronectin, Smoothened, FGFR1, OSF-2, SUFU, CD147, HGF, PTCH1, HIF1A, STK36, FGF2, SHH, GLI-1, IGF-1, HGF receptor (Met)
8	Development_Negative regulation of STK3/4 (Hippo) pathway and positive regulation of YAP/TAZ function	9.25E-07	ASPP1, LARG, G-protein alpha-q/11, Angiotensin II, YAP1 (YAp65), MLCP (reg), LATS1, ARHGEF2, Nephrocystin-4, FRMD4A, STK4, Itch, Actin cytoskeletal, TAZ, PDK (PDPK1), RhoA, G-protein alpha-12 family, PARD3, Citron, LPAR1, LIMD1, ASPP2, RhoGAP5, KIBRA, S1P2 receptor, NEDD4, ILK, MOBKL1A, WW45, PDZ-RhoGEF, Mol1b, JNK(MAPK8-10)
9	Development_Embryonal epaxial myogenesis	9.95E-07	FZD6, Lef-1, KIF27, Smoothened, MYOG, PAX3, SUFU, PTCH1, Myostatin, STK36, SHH, WNT1, GLI-3, HES1, WNT, Pitx2, GLI-1, Frizzled, WNT6, MYOD
10	Role of tumor-infiltrating B cells in anti-tumor immunity	1.02E-06	IL-18R1, MLANA, IP10, CD20, CTAG2, JAK1, DHFR, MAGE-1 antigen, POLR2B, Apo-2L(TNFSF10), IFN-alpha/beta receptor, Dsk2 (ubiquilin-2), ADAM-TS9, MAGE-3, IFN-alpha, GAGE2, CD27(TNFRSF7), MHC class I, MAGE-4 antigen, NXF2, MAGEB2, NF-kB, CT47A, SDF-1, CXCR4, IL-2, T-bet, G-protein alpha-i family, AID, IL4RA, IRF4, JAK3, Btk, NY-ESO-1, XAGE1, CXorf61, SOX2, FasR(CD95), KTN1, MAGEC2, MAGEA10, BLIMP1 (PRDI-BF1)
11	Immune response_IFN-alpha/beta signaling via PI3K and NF-kB pathways	1.03E-06	Cyclin D3, JAK1, RPS6, DHFR, NMI, eIF4E, CDC25A, Apo-2L(TNFSF10), IFN-alpha/beta receptor, p21, IFNAR1, I-kB, PI3K reg class IA (p85), GSK3 beta, NF-kB2 (p100), Rb protein, IFN-alpha, p19, CREB1, p130, MEK1/2, RSAD2, b-Myb, NF-kB, AKT(PKB), PDK (PDPK1), p107, p27KIP1, RelA (p65 NF-kB subunit), NF-kB2 (p52), TRAF2, E2F1, IFNAR2, p90RSK1, eIF4G1/3, PCNA, GBP1, Cyclin E, eIF4A, FOXO3A, p15, MNK2(GPRK7), Cyclin A
12	E-cadherin signaling and its regulation in gastric cancer	1.11E-06	DNMT3B, FGFR2, FGF1, Alpha-actinin, Dsh, Actin, Ubiquitin, GSK3 beta, SIP1 (ZFHX1B), HGF, RhoA, Formin, IQGAP1, WNT, SLUG, SNAIL1, Plakoglobin, HAKAI, HGF receptor (Met), p120-catenin, BMP2, Frizzled
13	Signal transduction_Angiotensin II signaling via Beta-arrestin	1.18E-06	eIF4E, 14-3-3, Angiotensin II, Beta-arrestin2, MLCP (reg), STAR, GSK3 beta, Itch, TRPV4, DGK, JNK3(MAPK10), AKT(PKB), MYLK1, p27KIP1, Clathrin heavy chain, MLCK, Casein kinase II, beta chain (Phosvitin), RhoA, CACNA1C, PP2A catalytic, Casein kinase II, alpha' chain (CSNK2A2), p90Rsk, ROCK, TRPC3, MRLC, Beta-adaptin 2, Clathrin, SSH1L, Cofilin, p23 co-chaperone
14	Role of activation of WNT signaling in the progression of lung cancer	1.32E-06	FZD6, Oct-3/4, SFRP2, Krm1, APC protein, Lef-1, DVL-1, TCF7L2 (TCF4), VEGF-A, p21, Dsh, WIF1, GSK3 beta, Survivin, DKK3, Axin2, SKP2, Axin, CD147, iNOS, Tcf(Lef),

			WNT3, ARD1, JNK1(MAPK8), SFRP5, WNT1, SFRP4, WNT2B, LKB1, WNT, WNT10B, WNT2, LRP6, SUZ12, HOXB9, p38 MAPK, Frizzled
15	c-Myc in multiple myeloma	1.41E-06	GRP78, eIF4E, YB-1, VEGF-A, HDAC6, p21, IL-6, hnRNP A1, HIF1A, IRF4, CD33, p38 MAPK, PTBP1
16	Development_Hedgehog signaling	1.75E-06	GAS1, SPOP, CDK11, KIF27, Smoothened, Beta-arrestin2, Ubiquitin, CDON, GSK3 beta, Itch, Skp2/TrCP/FBXW, DHH, HSP90, SUFU, PTCH1, MLK2(MAP3K10), STK36, GLI-3R, SHH, DYRK2, GLI-3, Casein kinase I, HIP, GLI-1, CDC37, PKA-cat (cAMP-dependent)
17	Defective macrophage-mediated bacterial phagocytosis in COPD	2.23E-06	MSR1, FCGR3A, HDAC6, C6orf134, CDC42, MANR, AKT(PKB), LRP1, Tubulin (in microtubules), SR-BI, Sirtuin2, RhoB, CD36, CR1, Tubulin alpha, C1qRp, NRF2
18	Immune response_IFN-alpha/beta signaling via MAPKs	2.7E-06	PML, IP10, JAK1, TCF7L2 (TCF4), Apo-2L(TNFSF10), IFN-alpha/beta receptor, p21, IFNAR1, VAV-1, PL scramblase 1, IKK-epsilon, Ubiquitin, TAP1 (PSF1), MAPKAPK2, IFN-alpha, MEK6(MAP2K6), Axin2, p130, RSAD2, AKT(PKB), p27KIP1, HIP-2, Lck, CD45, MEK3(MAP2K3), IFNAR2, JNK1(MAPK8), MAPKAPK3, AP-1, Filamin B (TABP), FOXO3A, FasR(CD95), TRIM6, JNK(MAPK8-10), p38 MAPK
19	DNA damage_ATM/ATR regulation of G2/M checkpoint: cytoplasmic signaling	3.46E-06	p38alpha (MAPK14), PP1-cat, 14-3-3, PP2A regulatory, CDC25A, MLCP (reg), TAO2, MAPKAPK2, MEK6(MAP2K6), GADD45 alpha, PLK1, Chk2, Histone H3, CDC25C, MEK3(MAP2K3), CDC25B, Nucleolysin TIAR, MARKK, PP2A catalytic, UBE2C, ATM, FOXO3A, Aurora-B, p38gamma (MAPK12), p38 MAPK, DCK, JIK
20	Signal transduction_AKT signaling	3.53E-06	RHEB2, Cyclin D3, GAB1, RPS6, p21, I-kB, Bax, PI3K reg class IA, GSK3 alpha/beta, NF-kB, AKT(PKB), HSP90, PDK (PDPK1), p27KIP1, Cyclin D, PP2A catalytic, Caspase-9, Hamartin, GYS1, PCNA, PTEN, FOXO3A, HGF receptor (Met), Bim
21	Chemotaxis_Lysophosphatidic acid signaling via GPCRs	3.66E-06	c-Fos, LARG, ROCK1, PRK1, PKC-zeta, G-protein alpha-q/11, p21, IP3 receptor, YAP1 (YAp65), MLCP (reg), LPAR4, PI3K reg class IA (p85), Bax, GSK3 beta, PLC-beta, DIA1, LPAR6, CREB1, MEK1/2, CDC42, Actin cytoskeletal, TAZ, IL13RA2, AKT(PKB), PDK (PDPK1), RhoA, G-protein alpha-12 family, LPAR1, Tcf(Lef), G-protein alpha-i family, F-Actin cytoskeleton, MKL2, Caspase-9, CD36, G-protein gamma 12, p130CAS, ROCK, PRKD1, AP-1, Caspase-7, PAK, PDZ-RhoGEF, PKC, FasR(CD95), FAK1, SIVA1, Rho GTPase, JNK(MAPK8-10), MKL1, ADAM17, p38 MAPK, Cofilin, PREX1
22	Transport_Clathrin-coated vesicle cycle	3.87E-06	VTI1A, Myosin I, Rab-8, GCC2, VTI1B, PI3K cat class III (Vps34), DAB2, Actin, YKT6, RAB9P40, RABGEF1, Rab-7, Syntaxin 5, TIP47, GDI2, Actin cytoskeletal, EEA1, Clathrin heavy chain, VAMP8, VAMP7, PIP5KIII, SAR1A, SNX9, VAMP4, Syntaxin 8, Syntaxin 7, GS15, SAR1, Endophilin B1,

			RILP (Rab interacting lysosomal protein), Rab-9, Rab11-FIP2, PREB, Clathrin
23	Transcription_HIF-1 targets	4.07E-06	PDK1, NIX, PDGF-B, EG-VEGF, PLGF, Oct-3/4, VEGF-A, Cyclin G2, 5'-NTD, ENO1, p21, PGK1, MDR1, ID2, FECH, F263, HXK2, WT1, ALDOC, Mcl-1, Epo, SDF-1, CXCR4, LRP1, PKM2, ARNT, DEC2, iNOS, ROR-alpha, CITED2, HIF1A, HIF-1, GPI, Endoglin, FGF2, IBP1, NANOG, ALDOA, SOX2, CX3CR1, ABCG2, HGF receptor (Met)
24	DNA damage_p53 activation by DNA damage	4.71E-06	P53AIP1, p38alpha (MAPK14), PML, 14-3-3, PP2A regulatory, p21, Bax, TTC5 (Strap), PP2A structural, MEK6(MAP2K6), GADD45 alpha, PUMA, RFWD3, PLK3 (CNK), Chk2, RelA (p65 NF-kB subunit), SMG1, E2F1, DAXX, AATF (Che-1), MARKK, PP2A catalytic, DYRK2, ELAVL1 (HuR), DDB2, USP7, ATM, JNK(MAPK8-10), p38 MAPK, PP2C gamma
25	Development_Muscle progenitor cell migration in hypaxial myogenesis	5.02E-06	GAB1, E2A, MEF2C, FGF8, SPRY1, FGFR1, MYOG, FGF4, PAX3, RBP-J kappa (CBF1), SDF-1, CXCR4, HGF, G-protein alpha-i family, SOS, HES1, SHP-2, MYOD/E47, SNAIL1, LBX1, HGF receptor (Met), ADAM17, MYOD
26	Cell cycle_The metaphase checkpoint	5.59E-06	CDC20, CENP-C, Aurora-C, CENP-F, MAD1 (mitotic checkpoint), Dynein 1, cytoplasmic, heavy chain, Survivin, PLK1, HP1 gamma, NSL1, CENP-B, CENP-H, INCENP, SPBC25, BUB3, Rod, CDCA1, Aurora-B, HP1 alpha, Zwilch, AF15q14
27	Development_TGF-beta receptor signaling	5.62E-06	Sno-N, eIF4E, GADD45 beta, FKBP12, p21, SMAD2, MEK6(MAP2K6), MEK2(MAP2K2), TSC-22, NF-kB, YY1, TAK1(MAP3K7), MEKK4(MAP3K4), Importin (karyopherin)-beta, SMURF1, TGF-beta receptor type II, Ski, SMURF2, MEK3(MAP2K3), SOS, SARA, NFKBIA, TAB1, p15, FAST-1/2, Caveolin-1, p38 MAPK
28	Cell adhesion_Tight junctions	6.08E-06	Rich1, JAM2, MUPP1, EPB41, F-Actin, ARP3, ACTR3, PKC-zeta, JAM1, Actin, APXL, Myosin VIIA, CDC42, Actin cytoskeletal, Tubulin (in microtubules), RhoA, PARD3, Myosin II, ROCK, Actomyosin, PDZ-RhoGEF, Tubulin alpha, PKC-lambda/iota, MRLC
29	Proteolysis_Role of Parkin in the Ubiquitin-Proteasomal Pathway	6.64E-06	FBXW7, UBCH7, Parkin, UBCH8, Caspase-8, SIAH1, HSP70, Tubulin beta, Alpha-synuclein, GPR37, Septin 5 (CDC-REL1), UBE1, Tubulin alpha, Cyclin E, UBC7, Synphilin 1
30	Development_PIP3 signaling in cardiac myocytes	7.11E-06	RHEB2, GAB1, RPS6, 14-3-3, PKC-zeta, PI3K reg class IA, Insulin receptor, GSK3 alpha/beta, CREB1, CDC42, AKT(PKB), PDK (PDPK1), HGF, G-protein alpha-12 family, PARD3, Cyclin D, SOS, Hamartin, p90Rsk, GYS1, PTEN, FOXO3A, IGF-1, HGF receptor (Met), PI3K reg class IB (p101)
31	Development_Thrombopoietin signaling via ERK1/2 and PI3K	7.94E-06	PDK1, Cyclin D3, c-Fos, GAB1, PP1-cat, Glycoprotein VI, PKC-zeta, CrkL, VEGF-A, GATA-1, p21, Thrombopoietin, USF1, PI3K reg class IA (p85), GSK3 beta, CREB1, MEK1/2, AKT(PKB), PDK (PDPK1), TAL1, p27KIP1, RelA (p65 NF-kB

			subunit), ITGA2B, c-Cbl, HIF1A, DNA-PK, SOS1, AML1 (RUNX1), SHP-2, FOXO3A, FLI1, p38 MAPK
32	Signal transduction_Angiotensin II/AGTR1 signaling via Notch, Beta-catenin and NF-kB pathways	8.45E-06	HES5, CCL2, TCF7L2 (TCF4), ROCK1, VEGF-A, TRPC6, Angiotensin II, Fibronectin, YAP1 (YAp65), I-kB, GSK3 beta, NF-kB2 (p100), Axin2, IL-6, NF-kB, AKT(PKB), RBP-J kappa (CBF1), PDK (PDPK1), TAK1(MAP3K7), RhoA, HEY1, RelA (p65 NF-kB subunit), NF-kB2 (p52), p90RSK1, PRKD1, PKC, HES1, WISP1, Connexin 43, HEY2, Angiotensinogen, SNAIL1, ADAM17, p38 MAPK, PKA-cat (cAMP-dependent)
33	Development_Role of growth factors in the maintenance of embryonic stem cell pluripotency	8.95E-06	FGFR2, c-Fos, GAB1, Oct-3/4, ROCK1, SMAD2, FGFR1, PI3K reg class IA (p85), GSK3 beta, PI3K reg class IA, IGF-2, MEK2(MAP2K2), AKT(PKB), PDK (PDPK1), Activin A, TGF-beta receptor type II, Myosin II, FGF2, SOS, Caspase-9, NANOG, ROCK, SOX2, SHP-2, ActRIIB, IGF-1, Bim
34	Development_Role of HDAC and calcium/calmodulin-dependent kinase (CaMK) in control of skeletal myogenesis	8.95E-06	p38alpha (MAPK14), HDAC9, ERK5 (MAPK7), Calcineurin A (catalytic), RHEB2, NF-AT1(NFATC2), MEF2C, 14-3-3, HDAC4, p21, CaMK IV, MYOG, PI3K reg class IA, IGF-2, MEK6(MAP2K6), AKT(PKB), PDK (PDPK1), CACNA1C, MEF2, HDAC5, NCOA2 (GRIP1/TIF2), CARM1, Calmodulin, MAP3K3, IGF-1, Calcineurin B (regulatory), MYOD
35	Development_YAP/TAZ-mediated co-regulation of transcription	9.73E-06	TEF-5, PML, TBX5, Oct-3/4, Lef-1, TEF-1, ID3, ID1, VEGF-A, SMAD2, YAP1 (YAp65), ID2, Survivin, Neurotractin, SIP1 (ZFHX1B), PAX3, TAZ, Catalase, PUMA, HIF1A, FOXM1, ErbB4(ICD), NANOG, TEF-4, TEF-3, SOX2, SLUG, SNAIL1
36	Development_Negative regulation of WNT/Beta-catenin signaling at the receptor level	1.02E-05	Sclerostin, AP complex 2 medium (mu) chain, DAB2, WIF1, CDON, Krm, Syndecans, Casein kinase II, GSK3 alpha/beta, SFRP, Axin, LRP1, SMURF1, Tcf(Lef), APCDD1, PEDF (serpinF1), LRP4, ZNRF3, WNT, LRP6, Clathrin, Glypican-6, AAK1, Frizzled
37	Development_Regulation of cytoskeleton proteins in oligodendrocyte differentiation and myelination	1.04E-05	CNTN1 (F3), CDK5R1 (p35), HDAC6, KLHL2, PDGF-R-alpha, Gelsolin, VAV-1, MLCP (reg), TPPP (p24), ERMN, CDC42, Actin cytoskeletal, Fyn, PDGF receptor, Tubulin (in microtubules), Tubulin beta, RhoA, p190RhoGAP, Myosin II, Tubulin alpha, MAP6, MAP4, PTPR-alpha, MRLC, FAK1, MAG, CDK5R2 (p39), MELC, Cofilin
38	Cell cycle_Start of DNA replication in early S phase	1.1E-05	ORC2L, MCM5, RPA3, MCM10, ORC6L, ORC4L, MCM2, CDC45L, CDC7, ORC1L, MCM4, E2F1, PP2A catalytic, Geminin, CDC18L (CDC6), Cyclin E, HP1 alpha, DRF1, Histone H1
39	Muscle contraction_GPCRs in the regulation of smooth muscle tone	1.19E-05	TRPC5, TRPC4, LARG, TRPC6, G-protein alpha-q/11, Angiotensin II, IP3 receptor, MLCP (reg), Prostacyclin receptor, PLC-beta, MLCK, RhoA, GEFT, G-protein alpha-12 family, Adenosine A2b receptor, ACM2, G-protein alpha-i family, CACNA1C, AVP extracellular region, Myosin II, OT, TBXA2R, MyHC, ACM3, Histamine H1 receptor, ROCK, PDZ-RhoGEF, G-protein alpha-s, Calmodulin, TRPC3,

			Alpha-1A adrenergic receptor, MRLC, Telokin, TRPC7, G-protein alpha-q, MELC, PKA-cat (cAMP-dependent)
40	Transcription_Role of heterochromatin protein 1 (HP1) family in transcriptional silencing	1.24E-05	HDAC9, HDAC4, CDC25A, HP1, MYOG, MBD2, Rb protein, HDAC2, Mi-2 alpha, HP1 gamma, Mi-2, SETDB1, MeCP2, Histone H3, E2F1, MEF2, HDAC5, HP1 beta, Cyclin E, HP1 alpha, Histone H4, MYOD
41	Stem cells_Pancreatic cancer stem cells in tumor metastasis	1.24E-05	LARG, VAV-1, IP3 receptor, G-protein alpha-13, MLCP (reg), CDC42, Fyn, SDF-1, MLCK, CXCR4, RhoA, G-protein alpha-i family, F-Actin cytoskeleton, Myosin II, MyHC, ROCK, PDZ-RhoGEF, Calmodulin, MRLC, FAK1, MELC, PI3K reg class IB (p101)
42	Oxidative stress_ROS-induced cellular signaling	1.26E-05	Casein kinase II, alpha chains, p38alpha (MAPK14), HES5, VEGF-A, TXNIP (VDUP1), p21, Bak, FASN, Bax, GSK3 beta, FTL, FTH1, IL-6, GADD45 alpha, NF-kB, AKT(PKB), Catalase, PUMA, Thioredoxin, PLK3 (CNK), Chk2, RelA (p65 NF-kB subunit), iNOS, IRP1, HIF1A, Pin1, Glutaredoxin 1, JNK1(MAPK8), NFKBIA, HSPA1A, ELAVL1 (HuR), PRKD1, PKC, LKB1, PTEN, HES1, ATM, DLC1 (Dynein LC8a), JNK(MAPK8-10), SAE2, NRF2, ADAM17, p38 MAPK, APEX, NALP3
43	Neurophysiological process_Dynein-dynactin motor complex in axonal transport in neurons	1.4E-05	DCTN1(p150Glued), Importin (karyopherin)-alpha, HDAC6, Vimentin, Ubiquitin, Centractins, DYNLL, Rab-7, Dynein 1, cytoplasmic, heavy chain, Alpha-centractin, DYNLT, HAP40, JNK3(MAPK10), PRNP, AKT(PKB), Snapin, MAPRPE1(EB1), Tubulin (in microtubules), Importin (karyopherin)-beta, Dynein 1, cytoplasmic, light chains, BPAG1, RILP (Rab interacting lysosomal protein), Kinesin heavy chain, Kinesin light chain, Carboxypeptidase H, BDNF, SPTBN2
44	NRF2 regulation of oxidative stress response	1.4E-05	Casein kinase II, alpha chains, CRM1, BACH1, SMRT, Ubiquitin, GSK3 beta, UGT1A1, SOD1, PI3K reg class IA, MafK, Actin cytoskeletal, Fyn, AKT(PKB), GSTA3, MafF, PDK (PDPK1), Casein kinase II, beta chain (Phosvitin), Thioredoxin, eIF2AK3, GCL reg, ENC1, PRDX1, JNK1(MAPK8), PKC, GCL cat, TXNRD1, NRF2
45	Anti-apoptotic action of Gastrin in pancreatic cancer	1.44E-05	p38alpha (MAPK14), Apaf-1, LARG, PI3K reg class IA (p85), Bax, MEK6(MAP2K6), AKT(PKB), PDK (PDPK1), PPAR-gamma, MEKK4(MAP3K4), RhoA, RelA (p65 NF-kB subunit), CCKBR, MEK3(MAP2K3), Caspase-9, NFKBIA, Annexin II, FOXO3A, FAK1, G-protein alpha-q
46	Cytoskeleton remodeling_Neurofilaments	1.45E-05	NEFL, CDK5R1 (p35), GFAP, DCTN1(p150Glued), Vimentin, Tubulin gamma 1, Actin cytoskeletal, Peripherin, Tubulin (in microtubules), Plectin 1, Tubulin beta, Tubulin gamma, MUNC18, BPAG1, Tubulin alpha, Kinesin heavy chain
47	Inhibition of Ephrin receptors in colorectal cancer	1.54E-05	c-Rel (NF-kB subunit), TCF7L2 (TCF4), Ephrin-A receptor 3, CDC42, Ephrin-B1, RhoA, Ephrin-A receptor 1, Ephrin-A receptors, Ephrin-B receptor 3, Ephrin-B receptor 1, ROCK,

			Ephrin-B, WNT, Ephrin-B receptor 2, FAK1, Ephrin-A, Ephrin-B receptors, Frizzled
48	Immune response_Fc epsilon RI pathway: Lyn-mediated cytokine production	1.62E-05	ERK5 (MAPK7), Calcineurin A (catalytic), c-Fos, NF-AT1(NFATC2), CCL2, MEF2C, BFL1, GATA-1, TSLP, VAV-1, IP3 receptor, Fc epsilon RI alpha, MEK6(MAP2K6), IL-13, IL-6, MEK1/2, CDC42, NF-kB, TAK1(MAP3K7), NF-AT2(NFATC1), IL-2, CCL1, MIST, IL-5, MEK3(MAP2K3), Fer, SOS, Fc epsilon RI beta, Btk, NFKBIA, NF-AT, AP-1, Calmodulin, PKC, IL-33, JNK(MAPK8-10), Bcl-10, p38 MAPK
49	Canonical WNT signaling pathway in colorectal cancer	1.68E-05	Galectin-3, SFRP2, APC protein, Lef-1, DVL-1, TCF7L2 (TCF4), VEGF-A, Axin1, p21, Dsh, WIF1, PI3K reg class IA (p85), GSK3 beta, Survivin, Axin2, CAS-L, Axin, iNOS, ENC1, Leptin receptor, SFRP5, WNT1, SFRP4, Mucin 2, WNT, SLUG, WNT2, LRP6, HGF receptor (Met), Frizzled, WNT6
50	Proteolysis_Putative ubiquitin pathway	1.94E-05	FBXW7, UBCH7, Parkin, UBCH8, Ubiquitin, UBCH6, SKP2, UEV1A, HSP70, GPR37, Septin 5 (CDC-REL1), UBE1, MJD (ataxin-3), RING-box protein 1, Synphilin 1

Table S5. Pathway analysis of LAGE2B-coexpressed genes from public breast cancer databases using the MetaCore database ($p<0.01$ set as the cutoff value)

#	Maps	pValue	Network Objects from Active Data
1	Immune response_IFN-alpha/beta signaling via PI3K and NF-kB pathways	3.69E-09	PI3K cat class IA, RelA (p65 NF-kB subunit), Cyclin D3, Tyk2, TRAF2, IRS-1, PDCD4, E2F1, RPS6, DHFR, NMI, eIF4E, ERK1/2, IFNAR2, eIF4B, CDC25A, p90RSK1, eIF4G1/3, IFN-alpha/beta receptor, NIK(MAP3K14), IFNAR1, PKC-epsilon, PCNA, 4E-BP1, I-kB, p70 S6 kinases, GSK3 beta, CDK4, IFN-alpha, p19, eIF4A, CREB1, p130, MEK1/2, Cyclin A, b-Myb, NF-kB, p107, p27KIP1, IL-12 beta
2	Development_PIP3 signaling in cardiac myocytes	1.33E-08	HGF, G-protein alpha-12 family, PI3K cat class IA, PARD3, RHEB2, GAB1, IRS-1, RPS6, Cyclin D, 14-3-3, PKC-zeta, PTP-1B, SOS, Hamartin, p90Rsk, BAD, 4E-BP1, PI3K reg class IA, PTEN, Insulin receptor, GSK3 alpha/beta, CREB1, IGF-1 receptor, G-protein beta/gamma, HGF receptor (Met)
3	Signal transduction_AKT signaling	4.43E-08	PI3K cat class IA, RHEB2, Cyclin D3, GAB1, IRS-1, RPS6, Cyclin D, PP2A catalytic, Hamartin, BAD, PCNA, 4E-BP1, I-kB, Bax, PI3K reg class IA, PTEN, MDM2, GSK3 alpha/beta, IGF-1 receptor, NF-kB, HGF receptor (Met), p27KIP1, Bim
4	Apoptosis and survival_Role of PKR in stress-induced apoptosis	6.07E-08	TRAF3, Tyk2, ATF-3, TRAF2, eIF4E, ERK1/2, ATF-4, PP2A regulatory, PP2A catalytic, IFN-alpha/beta receptor, NFKBIA, PACT, 4E-BP1, I-kB, TARBP2, IFN-alpha, IKK-beta, NFAT-90, PPP2R5A, NFKBIB, IRF3, NF-kB, MSK2, TAB2, NF-kB p50/p65, TLR3
5	DNA damage_ATM/ATR regulation of G1/S checkpoint	7.8E-08	Chk2, hnRNP K, SMG1, FBXW7, ERK1/2, Histone H2AX, PP2A regulatory, CDC25A, PP2A catalytic, BTG2, PER3, PCNA, p70

			S6 kinases, CDK4, PP2A structural, ATM, MEK2(MAP2K2), MDM2, c-Abl, Cyclin A, Cyclin D1, FBXO31, p27KIP1
6	Immune response_IL-11 signaling pathway via MEK/ERK and PI3K/AKT cascades	8.21E-08	gp130, RelA (p65 NF-kB subunit), ICAM1, IL-8, RPS6, ATF-1, ERK1/2, Leukocyte elastase, SOS, IL-11 receptor, p90Rsk, NFKBIA, YAP1 (YAp65), IL11RA, I-kB, p70 S6 kinases, PI3K cat class IA (p110-alpha), GSK3 beta, sIL11-RA, NFKBIB, CREB1, IL-6, MEK1/2, Fyn, Cyclin D1, SNAIL1, SFK, p27KIP1, IL-12 beta, IL-2
7	Development_Positive regulation of WNT/Beta-catenin signaling in the cytoplasm	1.78E-07	GSKIP, TBL1X, TGF-beta 1, IRS-1, RIPK4, APC protein, Alpha-1 catenin, 14-3-3, PKA-reg type II (cAMP-dependent), PP2A catalytic, RNF220, ZBED3, Beta-arrestin2, YAP1 (YAp65), PPP2R2A, Trabid, Tankyrases, USP9X, USP25, WNT, Insulin receptor, USP7, Jouberin, GSK3 alpha/beta, PP2C alpha, IGF-1 receptor, TBLR1, ERK2 (MAPK1), NKD2, Axin, MITF, SET7
8	Activation of TNF-alpha-dependent pro-tumoral effect in colorectal cancer	2.03E-07	RelA (p65 NF-kB subunit), IP10, NF-kB1 (p50), ICAM1, CCL2, MADD, TRAF2, IL-8, ERK1/2, ALDR, TRADD, SOS, NIK(MAP3K14), I-kB, GRO-1, NF-kB p65/c-Rel, TRAF1, IKK-beta, IL-6, MEK1/2, NF-kB, NF-kB p50/p65, p38 MAPK, Axin
9	Development_Delta- and kappa-type opioid receptors signaling via beta-arrestin	3.04E-07	Metenkefalin, GRK6, G-protein alpha-i family, GRK3, Kappa-type opioid receptor, Beta-arrestin2, Dynamin-1, PKC, GRK5, CREB1, G-protein beta/gamma, Clathrin, Leu-enkephalin, p27KIP1, Histone H4
10	IGF family signaling in colorectal cancer	3.2E-07	RelA (p65 NF-kB subunit), IRS-1, IL-8, E2F1, eIF4E, ERK1/2, Clusterin, SOS, IGF-2 receptor, Rad51, GIPC, 4E-BP1, I-kB, PI3K cat class IA (p110-alpha), ZNF143, GSK3 beta, PTEN, c-Myb, GSK3 alpha/beta, IGF-1 receptor, MEK1/2, Cyclin D1, NF-kB, ERK2 (MAPK1), ERK1 (MAPK3), IBP, MAT2A
11	Skeletal muscle atrophy in COPD	3.8E-07	ALK-4, KCRM, RelA (p65 NF-kB subunit), iNOS, RHEB2, MLC1F, ERK1/2, 14-3-3, MuRF1, eIF3S5, beta-MHC, SMAD2, MyHC, NEDD4, NFKBIA, MYOG, Myosin-IIA, 4E-BP1, I-kB, GSK3 beta, MLC2, Beta TnTF, ERK2 (MAPK1), NF-kB p50/p65, p38 MAPK, MYOD
12	Transport_Clathrin-coated vesicle cycle	3.92E-07	VTI1A, AP180, VAMP8, Myosin I, Rabaptin-5, HIP1, Rab11-FIP1, GOS-28, SAR1A, VTI1B, SNX9, PI3K cat class III (Vps34), VAMP4, Actin, YKT6, GS15, Rabenosyn-5, Rab-5A, RABGEF1, Myosin VI, SAR1, Rab-11A, RILP (Rab interacting lysosomal protein), Epsin 1, Syntaxin 5, Rab11-FIP2, PREB, EEA1, Clathrin, Clathrin heavy chain
13	Neurogenesis_NGF/ TrkA MAPK-mediated signaling	4.58E-07	GAB2, MATK, Efs/Sin, RIN, NF-kB1 (p50), SAC, MEK3(MAP2K3), ERK1/2, HB-EGF, PKC-zeta, CrkL, EGR1, SGK1, PP2A regulatory, PKA-reg (cAMP-dependent), SOS, PP2A catalytic, FRS2, C3G, PKC-epsilon, SH2B, p90Rsk, TY3H, NGF, MAPKAPK2, Calmodulin, MAGI-2, KIDINS220, CREB1, MEK1/2, SUR-8, Cyclin D1, RASGRF1, SP1, RGS2, p107, RIT, p38 MAPK, KCTD11
14	Immune response_IL-33 signaling pathway	5.79E-07	PI3K cat class IA, RelA (p65 NF-kB subunit), ICAM1, CCL2, IL-8, Histone H2B, MEK3(MAP2K3), ERK1/2, ST2L, NIK(MAP3K14), Ubiquitin, I-kB, GRO-1, PI3K reg class IA,

			ATF-2, GM-CSF, IL-33, IKK-beta, IRAK1, eNOS, IL-6, MEK1/2, NF-kB, TAB2, NF-kB p50/p65, Histone H2A
15	Immune response_IL-2 signaling via ERK, PI3K, and PLC-gamma	8.04E-07	GAB2, PI3K cat class IA, Lck, Cyclin D3, c-Cbl, IRS-1, E2F1, RPS6, ERK1/2, CrkL, SOS1, JAK3, 4E-BP1, p70 S6 kinases, Sirtuin1, PKC-theta, CDK4, c-Myb, MEK1/2, Cyclin A, Fyn, PYGM, NF-kB, ERK2 (MAPK1), ALPHA-PIX, p70 S6 kinase2, p27KIP1, ERK1 (MAPK3), Bim, IL-2
16	Apoptosis and survival_APRIL and BAFF signaling	8.26E-07	RelA (p65 NF-kB subunit), Calcineurin A (catalytic), TRAF3, NF-kB1 (p50), NF-AT1(NFATC2), TRAF2, BAFF-R, MIP-1-beta, BFL1, NIK(MAP3K14), CD21, TRAF5, NF-kB p50/p50, I-kB, IKK-beta, BCMA(TNFRSF17), TACI(TNFRSF13B), Cyclophilin B, CD23, NF-kB p50/p65
17	Oxidative stress_ROS-induced cellular signaling	1.05E-06	NOTCH1 (NICD), Thioredoxin, PLK3 (CNK), Chk2, ACACA, RelA (p65 NF-kB subunit), iNOS, IL-8, ERK1/2, EGR1, PKA-reg (cAMP-dependent), Pin1, NIK(MAP3K14), Bak, Cytochrome c, E2I, NFKBIA, HSPA1A, Bax, Sirtuin1, GSK3 beta, GPX1, PKC, LKB1, PTEN, FTH1, ATM, IKK-beta, DLC1 (Dynein LC8a), MDM2, c-Abl, IL-6, Cyclin D1, NF-kB, SP1, NF-kB p50/p65, PUMA, ADAM17, p38 MAPK
18	Development_Notch Signaling Pathway	1.25E-06	NOTCH1 (NICD), NOTCH1 receptor, Histone H3, c-Rel (NF-kB subunit), FBXW7, MAML1, GCN5, Jagged2, TLE, NFKBIA, Ubiquitin, p63, p73, HDAC2, Radical fringe, NOTCH1 precursor, HEY2, NOTCH1 (NEXT), ADAM17, Histone H4, MYOD
19	DNA damage_p53 activation by DNA damage	1.29E-06	PLK3 (CNK), Chk2, RelA (p65 NF-kB subunit), PML, SMG1, E2F1, DAXX, 14-3-3, PP2A regulatory, MARKK, PP2A catalytic, 14-3-3 theta, Tip60, Bax, Sirtuin1, TTC5 (Strap), PP2A structural, USP7, ATM, MDM2, c-Abl, PIG3, FBXO31, PUMA, p38 MAPK, PP2C gamma
20	PDE4 regulation of cyto/chemokine expression in inflammatory skin diseases	1.48E-06	RelA (p65 NF-kB subunit), iNOS, IP10, NF-kB1 (p50), NF-AT1(NFATC2), CCL2, IL-8, G-protein alpha-i family, ERK1/2, 14-3-3, PKA-reg (cAMP-dependent), NFKBIA, NF-kB p50/p50, G-protein alpha-i1, MAPKAPK2, Adenylate cyclase, CREB1, IL-6, MEK1/2, NF-kB p50/p65, p38 MAPK, IL-12 beta, IL-2
21	Development_MAG-dependent inhibition of neurite outgrowth	1.52E-06	NGFR(TNFRSF16), TrkC, Destrin, Myosin II, NT-4/5, Reticulon 4, MyHC, RhoGDI alpha, MLCP (reg), NGF, NGFR (ICD), RTN4R, NGFR (CTF), MRLC, RASGRF1, p200RhoGAP, MELC, ADAM17, BDNF
22	Signal transduction_Angiotensin II signaling via Beta-arrestin	1.57E-06	GRAF2, GRK6, eIF4E, ERK1/2, 14-3-3, CACNA1C, PP2A catalytic, p90Rsk, Beta-arrestin2, BAD, MLCP (reg), p70 S6 kinases, GSK3 beta, AP-2 alpha subunits, GRK5, MRLC, Beta-adaptin 2, TRPV4, DGK, Clathrin, MYLK1, p27KIP1, Clathrin heavy chain, SSH1L, MLCK
23	IL-17 and IL-17F-induced inflammatory signaling in normal and asthmatic airway epithelium	1.59E-06	IP10, ICAM1, IL-8, ERK1/2, ENA-78, p90Rsk, I-kB, GRO-1, GCP2, GM-CSF, MEK2(MAP2K2), CREB1, IL-6, NF-kB p50/p65, G-CSF, p38 MAPK

24	Development_BMP signaling in cardiac myogenesis	1.61E-06	BMP5, BMP receptor 2, MEK3(MAP2K3), BMP7, beta-MHC, CSX (Nkx2.5), BNP, alpha-MHC, ID2, ACTA1, MLC2, BMP6, ATF-2, BMPR1A, Cyclin D1, p38 MAPK, N-Myc, BMP2
25	Immune response_Histamine H1 receptor signaling in immune response	1.74E-06	RelA (p65 NF-kB subunit), Calcineurin A (catalytic), iNOS, ICAM1, MMP-1, IL-8, G-protein alpha-q/11, NFKBIA, I-kB, Calmodulin, PLC-beta, cPLA2, GM-CSF, IKK-beta, MEK2(MAP2K2), eNOS, IL-6, G-protein beta/gamma, ERK2 (MAPK1), NF-kB p50/p65, p38 MAPK, ERK1 (MAPK3)
26	Development_Hedgehog signaling	1.74E-06	Cul3/SPOP/Rbx1 E3 ligase, SPOP, Sin3A, PTCH1, MLK2(MAP3K10), STK36, GLI-3R, KIF27, Beta-arrestin2, GLI-2, Ubiquitin, CDON, BOC, GSK3 beta, GLI-3, Casein kinase I, Skp2/TrCP/FBXW, DHH, GLI-1, CDC37, Cullin 3, NUMB
27	Putative role of Estrogen receptor and Androgen receptor signaling in progression of lung cancer	2.32E-06	CYP1B1, RHEB2, G-protein alpha-i family, ERK1/2, 14-3-3, SOS, Cytochrome c, ESR2 (nuclear), NCOA2 (GRIP1/TIF2), ESR2 (mitochondrial), ESR2 (membrane), Kallikrein 3 (PSA), BAD, Androgen receptor, ID2, Bax, MAPKAPK2, MEK2(MAP2K2), CYP19, CREB1, Cyclin D1, NCOA1 (SRC1), ERK2 (MAPK1), p38 MAPK, ERK1 (MAPK3)
28	Transport_RAN regulation pathway	3.49E-06	Importin (karyopherin)-beta, CRM1, SUMO-1, RanBP1, NUP153, Importin (karyopherin)-alpha, RCC1, RanGAP1, NUP62, RanBP2, E2I, NUP58
29	Apoptosis and survival_Anti-apoptotic TNFs/NF-kB/Bcl-2 pathway	3.68E-06	NGFR(TNFRSF16), RelA (p65 NF-kB subunit), TRAF3, RANKL(TNFSF11), TRAF2, PKC-zeta, BFL1, TRADD, NIK(MAP3K14), TRAF5, FN14(TNFRSF12A), RANK(TNFRSF11A), NGF, I-kB, IKK-beta, BCMA(TNFRSF17), TACI(TNFRSF13B), IRAK1/2, NF-kB, CD40L(TNFSF5)
30	Development_Negative regulation of WNT/Beta-catenin signaling in the cytoplasm	4.4E-06	Prickle-1, NOTCH1 receptor, c-Cbl, CXXC4, E2F1, RIPK4, APC protein, Alpha-1 catenin, FAF1, Casein kinase I epsilon, PP2A catalytic, PI3K cat class III (Vps34), SENP2, RNF185, Casein kinase I alpha, YAP1/TAZ, YAP1 (YAp65), G-protein alpha-13, WWP1, LATS1, STK4, WNT, GSK3 alpha/beta, G-protein beta/gamma, WDR26, Skp2/TrCP/FBXW, TAZ, Cyclin D1, NKD2, DACT3, Axin
31	Development_Thrombopoietin signaling via ERK1/2 and PI3K	4.43E-06	GAB2, PI3K cat class IA, RelA (p65 NF-kB subunit), Cyclin D3, GAB1, ITGA2B, c-Cbl, NF-kB p50/c-Rel, DNA-PK, ERK1/2, PKC-zeta, CrkL, EGR1, PDHA (somatic), SOS1, Thrombopoietin, C3G, 4E-BP1, NF-E2 (45 kDa), GSK3 beta, CREB1, MEK1/2, SP1, FLI1, TAL1, p27KIP1, p38 MAPK
32	Immune response_IL-17 signaling pathways	4.88E-06	PI3K cat class IA, CCL20, iNOS, ICAM1, CCL2, RANKL(TNFSF11), MMP-1, IL-8, MEK3(MAP2K3), ERK1/2, ENA-78, NIK(MAP3K14), I-kB, GSK3 beta, GRO-1, PI3K reg class IA, GCP2, GM-CSF, IKK-beta, MEK2(MAP2K2), IL-6, NF-kB, SP1, G-CSF, p38 MAPK
33	CHDI_Correlations from Replication data_Causal network (positive correlations)	5.62E-06	PI3K cat class IA, IL-1 alpha, Calcineurin A (catalytic), Lck, NF-AT1(NFATC2), ICAM1, HIP1, MEK3(MAP2K3), PSMC2, CD28, MSK1/2 (RPS6KA5/4), MEF2, NIK(MAP3K14), LAT, RhoGDI alpha, I-kB, PKC-theta, Calmodulin, ZAP70, NR2,

			IKK-beta, CREB1, G-protein beta/gamma, PSD-95, IRAK1/2, CD83, NF-kB, HSP70, p38 MAPK, MEKK4(MAP3K4)
34	Transcription_Epigenetic regulation of gene expression	6.05E-06	SMYD2, GASC1, Histone H3, UTX, Histone H2B, GCN5, PRMT1, HDAC6, Sirtuin6, DOT1, RBB2, HBOA, Tip60, MLL1 (HRX), CARM1, Sirtuin1, DNMT3L, HDAC2, MYST1, MORF, Histone H2A, AOF1, Histone H4, SET7
35	PI3K signaling in gastric cancer	6.19E-06	HGF, PI3K cat class IA, RelA (p65 NF-kB subunit), IRS-1, CCKBR, IL-8, ErbB3, CBL-B, G-protein alpha-q/11, MDR1, BAD, I-kB, PI3K cat class IA (p110-alpha), GSK3 beta, PI3K reg class IA, PTEN, Cyr61, Cyclin D1, PRNP, NF-kB p50/p65, HGF receptor (Met), BMP2
36	Role of tumor-infiltrating B cells in anti-tumor immunity	6.28E-06	G3P2, IP10, CD20, MAGEC1, GAS11, T-bet, DHFR, G-protein alpha-i family, MAGE-1 antigen, CXCR5, POLR2B, IFN-alpha/beta receptor, IL4RA, IRF4, CD19, JAK3, Btk, ADAM-TS9, MAGE-3, ATF-2, IFN-alpha, GAGE2, KTN1, G-protein beta/gamma, NXF2, MAGEB2, NF-kB, CT47A, MAGEC2, CD23, IL-12 beta, IL-2, CD40L(TNFSF5)
37	ERBB family and HGF signaling in gastric cancer	7.47E-06	HGF, PI3K cat class IA, IL-8, MEK3(MAP2K3), ERK1/2, ErbB3, HB-EGF, EGR1, ErbB4, SOS, BAD, Amphiregulin, GSK3 beta, PI3K reg class IA, MEK1/2, Cyclin D1, TGF-alpha, ERK2 (MAPK1), SP1, HGF receptor (Met), p27KIP1, p38 MAPK, ERK1 (MAPK3)
38	NF-AT signaling in cardiac hypertrophy	7.79E-06	gp130, PI3K cat class IA, Calcineurin A (catalytic), GAB1, IRS-1, G-protein alpha-i family, ADSSL1, MYBPC3, HDAC5, G-protein alpha-q/11, beta-MHC, PKC-epsilon, CSX (Nkx2.5), Troponin I, cardiac, alpha-MHC, MEF2D, GSK3 beta, Calmodulin, PI3K reg class IA, LIF, IL-6, IGF-1 receptor, G-protein beta/gamma, Beta-1 adrenergic receptor, Troponin T, cardiac, LIF receptor
39	Aberrant production of IL-2 and IL-17 in SLE T cells	8.68E-06	NOTCH1 (NICD), TGF-beta 1, RelA (p65 NF-kB subunit), Lck, NF-AT1(NFATC2), Fc epsilon RI gamma, ROR-gamma, ERK1/2, PP2A cat (beta), PKA-reg (cAMP-dependent), SOS, LAT, I-kB, Calmodulin, ZAP70, NOTCH1 precursor, CREB1, IL-6, MEK1/2, CD3 epsilon, SP1, NF-kB p50/p65, p70 S6 kinase2, IL-2
40	Signal transduction_PKA signaling	9.17E-06	G-protein alpha-12 family, PDE3B, G-protein alpha-i family, PHK gamma, KDELR, PP2A regulatory, PKA-reg type II (cAMP-dependent), PKA-reg (cAMP-dependent), p90RSK1, PDE3A, Troponin I, cardiac, AKAP8, NFKBIA, G-protein alpha-13, BAD, Androgen receptor, Adenylate cyclase, GSK3 alpha/beta, NFKBIB, CREB1, GABA-A receptor beta-2 subunit, PKI
41	Immune response_BAFF-induced signaling	9.17E-06	RHEB2, TRAF2, RPS6, BAFF-R, MEK3(MAP2K3), eIF4E, ERK1/2, TRIM2, PI3K cat class IA (p110-delta), BAD, Ubiquitin, 4E-BP1, MAPKAPK2, GSK3 alpha/beta, BCMA(TNFRSF17), CREB1, MEK1/2, TACI(TNFRSF13B), ERK2 (MAPK1), p38 MAPK, ERK1 (MAPK3), Bim

42	Cell cycle_Nucleocytoplasmic transport of CDK/Cyclins	9.43E-06	CRM1, Cyclin D3, Cyclin D, Importin (karyopherin)-alpha, Karyopherin beta 1, GSK3 beta, CDK4, Cyclin A, Cyclin D1, ERK1 (MAPK3)
43	Development_Muscle progenitor cell migration in hypaxial myogenesis	1.06E-05	NOTCH1 (NICD), HGF, NOTCH1 receptor, GAB1, E2A, G-protein alpha-i family, MAML1, ERK1/2, SOS, FRS2, FGFR1, MYOG, G-protein beta/gamma, MYOD/E47, SNAIL1, NOTCH1 (NEXT), HGF receptor (Met), ADAM17, MYOD
44	Development_Negative regulation of WNT/Beta-catenin signaling in the nucleus	1.06E-05	KDM2, TBL1X, TRRAP, Calcineurin A (catalytic), c-Cbl, HBP1, Oct-3/4, CDX2, E2F1, APC protein, SOX17, Alpha-1 catenin, 14-3-3, Casein kinase I epsilon, TRIM33, GLI-3R, CHD8, TLE, SENP2, NF-AT5, GSK3 beta, HDAC2, WNT, eNOS, HIC1, CtBP, TBLR1, TAB2, HIC5, PPAR-gamma, Axin, Histone H1
45	Cell cycle_Regulation of G1/S transition (part 1)	1.23E-05	PLK3 (CNK), Chk2, TGF-beta 1, Cyclin D3, Cyclin D, PP2A regulatory, CDC25A, PP2A catalytic, SMAD2, Ubiquitin, GSK3 beta, CDK4, Cyclin A, Skp2/TrCP/FBXW, Cyclin D1, RING-box protein 1, SP1, p27KIP1
46	TNF-alpha-induced inflammatory signaling in normal and asthmatic airway epithelium	1.23E-05	CCL17, IP10, ICAM1, CCL2, IL-8, ERK1/2, PP2A catalytic, TSLP, NFKBIA, FN14(TNFRSF12A), I-kB, GRO-1, GM-CSF, IKK-beta, IL-6, NF-kB, NF-kB p50/p65, p38 MAPK
47	Immune response_IL-1 signaling pathway	1.34E-05	JAM2, PI3K cat class IA, IL-1 alpha, RelA (p65 NF-kB subunit), iNOS, NF-kB1 (p105), IP10, NF-kB1 (p50), ICAM1, CCL2, RANKL(TNFSF11), MMP-1, IL-8, MEK3(MAP2K3), ERK1/2, PKC-zeta, EGR1, NIK(MAP3K14), I-kB, MAPKAPK2, GRO-1, GM-CSF, IRAK1, IL-6, MEK1/2, Collagen II, NF-kB, NF-kB p50/p65, MYLK1, PPAR-gamma
48	Signal transduction_Adenosine A2A receptor signaling pathway	1.34E-05	RelA (p65 NF-kB subunit), Kif2a, NF-kB1 (p50), DNA-PK, ERK1/2, PKA-reg (cAMP-dependent), SOS, Kir6.2, SK4/IK1, I-kB, GSK3 beta, Adenylate cyclase, ATM, eNOS, CREB1, MEK1/2, SFK, p38 MAPK, IL-12 beta, Adenosine A2a receptor, Dopamine D2 receptor, IL-2
49	Interleukins-induced inflammatory response in asthmatic airway fibroblasts	1.42E-05	GRO-2, IL-1 alpha, RelA (p65 NF-kB subunit), ICAM1, CCL2, IL-8, ERK1/2, NGF, GRO-1, GM-CSF, IL-33, IL-6, MEK1/2, NF-kB, NF-kB p50/p65, G-CSF, p38 MAPK
50	Th2 cytokine- and TNF-alpha-induced inflammatory response in asthmatic airway fibroblasts	1.42E-05	TGF-beta 1, RelA (p65 NF-kB subunit), ICAM1, CCL2, IL-8, IL-4R type II, ERK1/2, Eotaxin-3, IL4RA, IL13RA1, GM-CSF, IL-6, NF-kB, NF-kB p50/p65, ITGA5, G-CSF, CD40L(TNFSF5)

Table S6. Pathway analysis of LAGE3-coexpressed genes from public breast cancer databases using the MetaCore database ($p<0.01$ set as the cutoff value)

#	Maps	pValue	Network Objects from Active Data
1	DNA damage_ATM/ATR regulation of G2/M checkpoint: cytoplasmic signaling	2.52E-13	p38alpha (MAPK14), BORA, PP1-cat, 14-3-3, PP2A regulatory, CDC25A, Nek11, CDK1 (p34), MLCP (reg), Chk1, MAPKAPK2, MEK6(MAP2K6), B56G, Cyclin B1, PLK1, MLCP (cat), Chk2, Brca1, Histone H3, CDC25C, beta-TrCP, MEK3(MAP2K3), CDC25B, JAB1, MARKK, PP2A catalytic,

			ATR, Aurora-A, MEK4(MAP2K4), UBE2C, MEKK1(MAP3K1), FOXO3A, c-Abl, Aurora-B, JNK2(MAPK9), Brca1/Bard1, p38 MAPK, DCK
2	Transcription_Negative regulation of HIF1A function	6.02E-13	FHL3, p14ARF, Casein kinase I delta, VHL, COMMD1 (MURR1), FBXW7, SART1, VCP, SKP1, UBXD7, Ubiquitin, GSK3 beta, EGLN2, MCM7, Elongin B, EAF2, HSP40, MCM2, HSP90, Calpain 1(mu), HSP70, HSPA4, SAT2, EGLN1, PLK3 (CNK), MCM3, DEC2, HSC70, PRDX2, CITED2, HIF1A, ARD1, PSMA7, Sirtuin2, HSP90 beta, AML1 (RUNX1), FHL1 (SLIM1), Cul2/Rbx1 E3 ligase, PTEN, Sirtuin7, SSAT, Elongin C, HIF-prolyl hydroxylase, PRDX4, CHIP, RUVBL2
3	Development_Negative regulation of WNT/Beta-catenin signaling in the nucleus	1.82E-12	ZNF703, TBL1X, Casein kinase I delta, Calcineurin A (catalytic), BACH1, HBP1, Oct-3/4, VHL, PGAM5, Alpha-1 catenin, TCF7L2 (TCF4), 14-3-3, Jade-1, Casein kinase I epsilon, Beta-catenin, PAX7, BCL9/B9L, PC1-CTT, TLE, Dsh, Menin, NF-AT5, GSK3 beta, HDAC2, ICAT, HIC1, LATS2, P15RS, CtBP, TBLR1, TAB2, PJA2, PPAR-gamma, Axin, TAK1(MAP3K7), SOX9, TRRAP, Tcf(Lef), CDX2, TRIM33, GLI-3R, CHD8, SENP2, Kaiso, SOX2, WNT, FOXO3A, GPX4, CHIBBY, HDAC1, Plakoglobin, RUVBL2, Frizzled, Histone H1
4	Development_Negative regulation of WNT/Beta-catenin signaling in the cytoplasm	1.93E-12	Casein kinase I delta, VHL, PP1-cat, RIPK4, Presenilin 1, Alpha-1 catenin, Laforin, FAF1, Casein kinase I epsilon, Beta-catenin, CYLD, PI3K cat class III (Vps34), DAB2, Dsh, Rac1, G-protein alpha-13, STK4, Itch, GSK3 alpha/beta, Ankyrin-G, LATS2, G-protein beta/gamma, WDR26, Skp2/TrCP/FBXW, TAZ, Axin, KLHL12, HECTD1, Prickle-1, HIPK2, Tcf(Lef), beta-TrCP, PP2A catalytic, PR72, SENP2, RNF185, YAP1/TAZ, MAP1LC3A, ELAVL1 (HuR), PEG3, Cul2/Rbx1 E3 ligase, Malin, PKC-alpha, Porf-2, WNT, Beclin 1, NEDD4L, Cyclin D1, NKD2, DACT3, Frizzled
5	Transcription_HIF-1 targets	3.81E-12	PDK1, NIX, TGF-beta 1, PDGF-B, EG-VEGF, PLGF, Oct-3/4, P4HA2, PFKL, REDD1, ENO1, Carbonic anhydrase IX, PGK1, Stanniocalcin 2, NIP3, MDR1, AK3, ID2, FECH, Adipophilin, F263, HXK2, PLAUR (uPAR), DEC1 (Stra13), Mcl-1, Epo, SDF-1, CXCR4, LRP1, PKM2, G3P2, TGF-beta 2, DEC2, Lysyl oxidase, ROR-alpha, Adrenomedullin, CITED2, HIF1A, GPI, Endoglin, FGF2, Angiopoietin 2, MGF, GLUT1, Carbonic anhydrase XII, ALDOA, SOX2, TGF-beta 3, CX3CR1, LOXL2, TGM2, Galectin-1, MCT4, ABCG2, HGF receptor (Met), Leptin
6	Development_Positive regulation of WNT/Beta-catenin signaling in the cytoplasm	3.83E-12	TBL1X, Bcl-9, TGF-beta 1, BIG1, IRS-2, IRS-1, UBE2B, PP1-cat, RIPK4, Alpha-1 catenin, 14-3-3, Beta-catenin, TGIF, Dsh, USP47, Rac1, Beta-arrestin2, CDK1 (p34), PPP2R2A, USP25, PR130, GSK3 alpha/beta, IGF-1 receptor, TBLR1, AKT(PKB), Axin, GSKIP, HECTD1, ITGB1, HIPK2, Tcf(Lef), 14-3-3 zeta/delta, TGT, PKA-reg type II (cAMP-dependent), PP2A catalytic, RNF220, ILK, Tankyrases, BIG2, USP9X, WNT, Jouberin, FAK1, PP2C alpha, JNK(MAPK8-10), SMAD3, NKD2, Frizzled

7	Chemotaxis_Lysophosphatidic acid signaling via GPCRs	7.37E-12	c-Fos, alpha-6/beta-1 integrin, PI3K cat class IA (p110-beta), H-Ras, LARG, ROCK1, PRK1, PKC-zeta, Beta-catenin, EGR1, HDAC7, G-protein alpha-q/11, PKC-epsilon, TRIP6, Rac1, IP3 receptor, LPAR2, MLCP (reg), LPAR4, PI3K reg class IA (p85), Bax, GSK3 beta, PLC-beta, FKHR, DIA1, LPAR6, Caspase-3, CREB1, MEK1/2, G-protein beta/gamma, CDC42, Actin cytoskeletal, N-CoR, TAZ, MSK1, AKT(PKB), PDK (PDPK1), c-Src, Tiam1, cPKC (conventional), G-protein alpha-12 family, PLC-eta 1, LPAR1, Tcf(Lef), G-protein alpha-i family, F-Actin cytoskeleton, MEK4(MAP2K4), 4E-BP1, p130CAS, ROCK, PRKD1, Caspase-7, PAK, p70 S6 kinase1, PKC, Vinculin, PLC-delta 1, Bcl-2, FasR(CD95), FAK1, SIVA1, Rho GTPase, JNK(MAPK8-10), MKL1, PLC-beta3, ADAM17, p38 MAPK, Elk-1, PREX1
8	Immune response_IFN-alpha/beta signaling via MAPKs	6E-11	PML, IP10, ISG15, Tyk2, TCF7L2 (TCF4), Beta-catenin, PRMT1, ZNF145, PIAS1, Apo-2L(TNFSF10), IFN-alpha/beta receptor, IFNAR1, IFN-beta, Rac1, Ubiquitin, TAP1 (PSF1), MAPKAPK2, PKC-theta, MEK6(MAP2K6), Axin2, p130, MSK1, AKT(PKB), p27KIP1, FZD7, HIP-2, Lck, CD45, PKR, MEK3(MAP2K3), IFNAR2, IRF9, MEK4(MAP2K4), Ku80, MAPKAPK3, MEKK1(MAP3K1), Filamin B (TABP), FOXO3A, FasR(CD95), IRF7, Cyclin D1, JNK(MAPK8-10), HDAC1, SMAD3, p38 MAPK
9	Transport_Clathrin-coated vesicle cycle	7.6E-11	NSF, VTI1A, AP180, Myosin I, Rabaptin-5, Rab-8, Eps15, VTI1B, PI3K cat class III (Vps34), DAB2, Dynamin-2, Actin, YKT6, RABGEF1, Syntaxin 5, Syntaxin 12, VPS45A, BIN1 (Amphiphysin II), TIP47, Actin cytoskeletal, EEA1, RABGDIA, Clathrin heavy chain, VAMP2, Syntaxin 6, Rab-4, PI3K reg class III (p150), VAMP8, HIP1, PIP5KIII, GOS-28, SAR1A, VAMP4, ARF1, Syntaxin 7, Rab-5A, SAR1, Rab-11A, Endophilin B1, Rab-9, Rab11-FIP2, Clathrin, Myosin Vb, Rip11
10	Oxidative stress_ROS-induced cellular signaling	7.81E-11	p38alpha (MAPK14), SREBP1 (nuclear), EGR1, PKA-reg (cAMP-dependent), TXNIP (VDUP1), Bak, Cytochrome c, FASN, E2I, Carbonic anhydrase IX, Bax, GSK3 beta, FTL, FTH1, IRP2, GRP75, NF-kB, AKT(PKB), NF-kB p50/p65, Cyclin B1, c-Src, Thioredoxin, PLK3 (CNK), Chk2, ACACA, RelA (p65 NF-kB subunit), KEAP1, Cul3/KEAP1/Rbx1 E3 ligase, Adrenomedullin, IRP1, HIF1A, SRX1, AMPK alpha subunit, Pin1, p300, HSPA1A, ELAVL1 (HuR), HSF1, PRKD1, p70 S6 kinase1, GPX1, PKC, PTEN, MEKK1(MAP3K1), HES1, HSP27, IKK-beta, DLC1 (Dynein LC8a), c-Abl, Cyclin D1, JNK(MAPK8-10), HIF-prolyl hydroxylase, HDAC1, SP1, NRF2, ADAM17, p38 MAPK, APEX, NALP3
11	Cell cycle_Spindle assembly and chromosome separation	1.89E-10	Cyclin B, Separase, Nek2A, CDC20, MAD2a, Importin (karyopherin)-alpha, RCC1, Kid, CSE1L, CDK1 (p34), Ubiquitin, Dynein 1, cytoplasmic, heavy chain, TPX2, Ran, Tubulin (in microtubules), Importin (karyopherin)-beta, NUMA1, ZW10, Dynein 1, cytoplasmic, light chains, Aurora-

			A, Securin, Tubulin alpha, KNSL1, Aurora-B, HEC, Dynein 1, cytoplasmic, intermediate chains
12	Translation_Regulation of EIF4F activity	2E-10	TGF-beta 1, RHEB2, IRS-1, H-Ras, eIF4E, PKC-zeta, Rac1, PI3K reg class IA, IGBP1, eIF4G2, MEK6(MAP2K6), MEK2(MAP2K2), CDC42, MSK1, AKT(PKB), PDK (PDPK1), TAK1(MAP3K7), MEK1(MAP2K1), Tiam1, PI3K cat class IA, TGF-beta receptor type II, MEK3(MAP2K3), eIF4B, SOS, PP2A catalytic, eIF4G1/3, PAK1, Hamartin, MEK4(MAP2K4), 4E-BP1, p70 S6 kinase1, MEKK1(MAP3K1), eIF4A, TAB1, p70 S6 kinase2, p38 MAPK
13	DNA damage_ATM/ATR regulation of G1/S checkpoint	4.35E-10	p38alpha (MAPK14), NFBD1, FBXW7, PP2A regulatory, CDC25A, PER3, p70 S6 kinases, Chk1, CDK4, PP2A structural, MEK2(MAP2K2), B56G, FBXO31, p27KIP1, Chk2, Brca1, SMG1, beta-TrCP, Histone H2AX, PP2A catalytic, BTG2, ATR, CDK2, ELAVL1 (HuR), PCNA, Cyclin E, FOXO3A, c-Abl, Cyclin A, Cyclin D1, Brca1/Bard1
14	Epithelial cell anoikis in COPD	6.42E-10	IRS-2, IRS-1, H-Ras, Alpha-actinin, alpha-6/beta-4 integrin, Bak, BAD, Talin, Bax, PI3K reg class IA, MEK6(MAP2K6), MMP-12, MEK1/2, Actin cytoskeletal, AKT(PKB), PDK (PDPK1), alpha-3/beta-1 integrin, PI3K cat class IA, MMP-1, ASK1 (MAP3K5), MEK3(MAP2K3), BPAG1, SOS, Beta-parvin, MKK7 (MAP2K7), ILK, MEK4(MAP2K4), Vinculin, Bcl-2, FAK1, Alpha-parvin, JNK(MAPK8-10), p38 MAPK, Bim
15	Development_Differentiation of white adipocytes	1.14E-09	p38alpha (MAPK14), SREBP1 (nuclear), BMP receptor 2, BMP4, H-Ras, FOXC2, HDAC3, SREBP1 precursor, UCP1, HIVEP2, FTase-alpha, MEK2(MAP2K2), CREB1, MEK1/2, PPARGC1 (PGC1-alpha), p107, PPAR-gamma, TAK1(MAP3K7), MEK1(MAP2K1), C/EBPbeta, SOS, p90RSK1, LIPS, p300, RIP140, PSAT, TAB1, BMPR1A, SMAD1, Leptin, BMP2, LPL, Perilipin, C/EBPalpha, C/EBPdelta
16	Anti-apoptotic action of Gastrin in pancreatic cancer	1.8E-09	p38alpha (MAPK14), Apaf-1, IRS-1, LARG, Cytochrome c, BAD, PI3K reg class IA (p85), Bax, FKHR, Caspase-3, MEK6(MAP2K6), AKT(PKB), Gastrin 17, PDK (PDPK1), Progastrin, c-Src, PPAR-gamma, MEKK4(MAP3K4), PI3K cat class IA, RelA (p65 NF-kB subunit), MEK3(MAP2K3), Annexin II, Bcl-2, FOXO3A, FAK1, G-protein alpha-q
17	Signal transduction_CXCR4 signaling via MAPKs cascades	2.96E-09	PDGF-B, c-Fos, IRS-1, EGR1, p90RSK2(RPS6KA3), G-protein alpha-i2, Rac1, Beta-arrestin2, G-protein alpha-13, BAD, Ubiquitin, N-Ras, CREB1, MEK1/2, G-protein beta/gamma, c-Src, SDF-1, CXCR4, MEK1(MAP2K1), RelA (p65 NF-kB subunit), NF-kB1 (p50), K-RAS, G-protein alpha-i family, ROCK, PAK, MEKK1(MAP3K1), CD3, CD3 zeta, ACKR3, JNK(MAPK8-10), SMAD3, p38 MAPK, Elk-1, PREX1
18	Signal transduction_IGF-1 receptor signaling pathway	3.8E-09	SREBP1 (nuclear), GAB1, IRS-2, IRS-1, H-Ras, eIF4E, PKC-zeta, FASN, BAD, Androgen receptor, I-kB, PI3K reg class IA (p85), GSK3 beta, FKHR, IGF-2, IGF-1 receptor, MEK1/2, AKT(PKB), PDK (PDPK1), PI3K cat class IA, RelA (p65 NF-kB subunit), ASK1 (MAP3K5), 14-3-3 zeta/delta, Cyclin D, SOS, MKK7

			(MAP2K7), MEK4(MAP2K4), 4E-BP1, 14-3-3 beta/alpha, p70 S6 kinase1, SHP-2, Bcl-2, FOXO3A, FAK1, MNK2(GPRK7), Bim
19	Cell cycle_Regulation of G1/S transition (part 1)	4.42E-09	TGF-beta 1, Cyclin D3, PP2A regulatory, CDC25A, Cyclin D2, Ubiquitin, GSK3 beta, CDK4, Skp2/TrCP/FBXW, p16INK4, p27KIP1, PLK3 (CNK), TGF-beta 2, Chk2, Brca1, TGF-beta receptor type II, beta-TrCP, Cyclin D, PP2A catalytic, CDK2, p70 S6 kinase1, Cyclin E, Cyclin A, Cyclin D1, RING-box protein 1, SMAD3, SP1
20	Cell cycle_The metaphase checkpoint	4.9E-09	Nek2A, CDC20, MAD2a, MAD2b, PMF1, CENP-C, CENP-E, BUB1, CENP-F, Dynein 1, cytoplasmic, heavy chain, Survivin, PLK1, HP1 gamma, MIS12, HZwint-1, ZW10, CENP-A, CENP-H, INCENP, SPBC25, Aurora-A, CDCA1, Aurora-B, HEC, HP1 alpha, AF15q14
21	G-protein signaling_Ras family GTPases in kinase cascades	5E-09	p38alpha (MAPK14), c-Fos, H-Ras, Rac1, p38beta (MAPK11), N-Ras, MEK2(MAP2K2), CDC42, MEKK4(MAP3K4), MEK1(MAP2K1), B-Raf, K-RAS, C/EBPbeta, MEK3(MAP2K3), PAK1, MEK4(MAP2K4), MEKK1(MAP3K1), JNK(MAPK8-10), R-Ras, p38 MAPK, Elk-1
22	Immune response_IFN-alpha/beta signaling via PI3K and NF-kB pathways	7.18E-09	Cyclin D3, ISG15, IRS-2, Tyk2, IRS-1, NMI, eIF4E, CDC25A, Apo-2L(TNFSF10), IFN-alpha/beta receptor, IFNAR1, PKC-epsilon, IFN-beta, CDK1 (p34), I-kB, p70 S6 kinases, PI3K reg class IA (p85), GSK3 beta, CDK4, EMSY, p19, CREB1, p130, MEK1/2, b-Myb, NF-kB, AKT(PKB), PDK (PDPK1), p16INK4, p107, p27KIP1, IFI17, PI3K cat class IA, RelA (p65 NF-kB subunit), I-TAC, IFNAR2, eIF4B, p90RSK1, eIF4G1/3, CDK2, PCNA, 4E-BP1, GBP1, PKC-alpha, Cyclin E, eIF4A, FOXO3A, IRF7, MNK2(GPRK7), Cyclin A
23	Immune response_B cell antigen receptor (BCR) pathway	7.54E-09	STIM1, Calcineurin A (catalytic), NCK1, c-Fos, c-Rel (NF-kB subunit), H-Ras, NF-kB p50/c-Rel, EGR1, BCAP, Fibronectin, CD19, Rac1, IP3 receptor, BAD, Cyclin D2, PI3K reg class IA (p85), ORAI1, GSK3 beta, CD79A, CDK4, FKHR, N-Ras, MEK6(MAP2K6), MEK2(MAP2K2), GSK3 alpha/beta, MEK1/2, CDC42, Actin cytoskeletal, NF-kB, AKT(PKB), NF-kB p50/p65, PDK (PDPK1), TAK1(MAP3K7), NF-AT2(NFATC1), MEKK4(MAP3K4), MEK1(MAP2K1), RelA (p65 NF-kB subunit), B-Raf, NF-kB1 (p50), K-RAS, PIP5KIII, MEK3(MAP2K3), CIN85, PP2A catalytic, SOS1, PIP5KI, p70 S6 kinase1, Calmodulin, CalDAG-GEFIII, CKLFSF7, MEKK1(MAP3K1), IKK-beta, PLC-gamma, Bcl-10, p38 MAPK, Elk-1
24	Neurogenesis_NGF/ TrkA MAPK-mediated signaling	8.78E-09	ERK5 (MAPK7), CDK5, c-Fos, APS, Fra-1, H-Ras, MEF2C, PKC-zeta, CrkL, EGR1, PP2A regulatory, PKA-reg (cAMP-dependent), DNAJA3 (TID1), VGF, PKC-epsilon, IP3 receptor, TY3H, MAPKAPK2, PLC-gamma 1, N-Ras, MEK6(MAP2K6), MAP2K5 (MEK5), KIDINS220, CREB1, MEK1/2, PLAUR (uPAR), MSK1, RUSC1 (NESCA), p107, c-Src, KCTD11, GAB2, B-Raf, NF-kB1 (p50), K-RAS, MEK3(MAP2K3), SOS, PP2A catalytic, C3G, SH2B, p90Rsk, p130CAS, Calmodulin, PKC-

			lambda/iota, SHP-2, SUR-8, Cyclin D1, SORBS1, SP1, JMJD3, SHB, p38 MAPK, Elk-1, FosB
25	Development_Positive regulation of WNT/Beta-catenin signaling in the nucleus	8.89E-09	SMYD2, TBL1X, Alpha-1 catenin, TCF7L2 (TCF4), FOXP1, USP5, Jade-1, Beta-catenin, BCL9/B9L, VCP, TLE, Dsh, UBR5, GSK3 beta, HDAC2, WIP1, RUNX, FOXK2, ICAT, TBLR1, SOX9, SOX11, Tcf(Lef), PIAS4, beta-TrCP, FOXM1, SHH, Pin1, p300, NCOA2 (GRIP1/TIF2), FAM53B, Kindlin-2, CARF, WNT, FOXO3A, HMGB2, HDAC1, APPL, RUVBL2, Frizzled
26	Transcription_Sin3 and NuRD in transcription regulation	9.32E-09	TR-alpha, RARbeta, SMRT, SAP130, RBBP4 (RbAp48), ARID4A, MBD2, HDAC2, NRSF, Mi-2 alpha, N-CoR, ARID4B, p66beta, PSF, MTA1, Mi-2, Histone H3, Sin3A, RXRA, RAR-beta/RXR-alpha, MTA2, SAP18, RBBP7 (RbAp46), HDAC1, SAP30, NRB54, p66alpha, Histone H4
27	Signal transduction_Angiotensin II signaling via Beta-arrestin	9.4E-09	GRK6, eIF4E, 14-3-3, Beta-arrestin2, BAD, MLCP (reg), Cytohesin2, p70 S6 kinases, GSK3 beta, PLC-gamma 1, Itch, DGK, AKT(PKB), c-Src, p27KIP1, Clathrin heavy chain, MLCK, MLCP (cat), MEK1(MAP2K1), Casein kinase II, beta chain (Phosvitin), GRAF2, ASK1 (MAP3K5), SET, AGTR1, CACNA1C, PP2A catalytic, p90Rsk, MEK4(MAP2K4), ROCK, GRK5, MRLC, Beta-adaptin 2, Clathrin, Beta-arrestin1, p23 co-chaperone
28	Cell cycle_Chromosome condensation in prometaphase	1.09E-08	TOP1, Cyclin B, CAP-G/G2, CAP-D2/D3, AKAP8, CDK1 (p34), TOP2, BRRN1, Histone H3, CAP-G, INCENP, Aurora-A, CAP-E, CAP-H/H2, Cyclin A, CNAP1, Aurora-B, Histone H1
29	Neutrophil resistance to apoptosis in COPD and proresolving impact of lipid mediators	1.39E-08	SAA1, TNF-R1 soluble, Apaf-1, FasR(CD95) soluble, gp91-phox, 14-3-3, TRADD, Bak, Cytochrome c, Caspase-8, p47-phox, BAD, Bax, PI3K reg class IA, Calpastatin, Caspase-3, MEK6(MAP2K6), RIPK1, FasL(TNFSF6), AKT(PKB), Calpain 1(mu), TNF-R1, Mcl-1, FPRL1, PI3K cat class IA, Cytochrome b-558, p22-phox, MEK3(MAP2K3), p90RSK1, MAP3K3, tBid, FasR(CD95), p40-phox, Smac/Diablo, p38 MAPK, Bid
30	Immune response_IL-3 signaling via JAK/STAT, p38, JNK and NF-kB	1.53E-08	STAT5A, MHC class II, DHA2, Cyclin D3, c-Fos, Tyk2, Granzyme B, H-Ras, SOCS1, ID1, Fibronectin, SPECC1, Rac1, BAD, Cyclin D2, I-kB, SRP9, Survivin, Cyclin A2, IL3RA, NF-kB, AKT(PKB), NF-kB p50/p65, Cyclin B1, c-Src, Mcl-1, P-selectin, IL-2R alpha chain, PKM2, Ephrin-B1, ITGB1, PI3K cat class IA, C/EBPbeta, MEK3(MAP2K3), RXRA, Bcl-6, MKK7 (MAP2K7), JAK3, MEK4(MAP2K4), IKK-beta, Bcl-2, Oncostatin M, STAT5, Cyclin D1, HDAC1, STAT6, Ephrin-B2, p38 MAPK, BMP2
31	Mechanisms of drug resistance in SCLC	1.57E-08	alpha-6/beta-1 integrin, Apaf-1, alpha-V/beta-1 integrin, Cytochrome c, Caspase-8, Fibronectin, Rad51, PKC-epsilon, MDR1, BAD, Bax, GSK3 beta, Survivin, PI3K reg class IA, Caspase-3, IGF-1 receptor, AKT(PKB), Collagen IV, HSP70, PDK (PDPK1), alpha-3/beta-1 integrin, ITGB1, Osteopontin, PI3K cat class IA, RelA (p65 NF-kB subunit), B-Raf, TERC, FGF2, CD9, MGF, HSPA1A, HSF1, p70 S6 kinase1, tBid, Bcl-2, FAK1, TOP2 alpha, Cyclin D1, p70 S6 kinase2, Bid

32	Cytoskeleton remodeling_Regulation of actin cytoskeleton organization by the kinase effectors of Rho GTPases	1.79E-08	Spectrin, PRK1, Alpha-actinin, RhoC, Rac1, Talin, MLCP (reg), GIT1, Cdc42 subfamily, MSN (moesin), ERM proteins, ARPC1B, RhoA-related, CDC42, Actin cytoskeletal, MLCK, MLCP (cat), BETA-PIX, RhoJ, F-Actin cytoskeleton, Myosin II, CPI-17, Alpha adducin, PAK1, PIP5KI, MyHC, Rac3, MRCK, ROCK, Actomyosin, PAK, Rac1-related, Vinculin, Rhov, MRLC
33	Immune response_Gastrin in inflammatory response	2.06E-08	GRO-2, p38alpha (MAPK14), ERK5 (MAPK7), c-Fos, IRS-1, H-Ras, LARG, MEF2C, G-protein alpha-q/11, PKC-epsilon, IP3 receptor, I-kB, PI3K reg class IA (p85), PLC-gamma 1, MEK6(MAP2K6), MAP2K5 (MEK5), MEK2(MAP2K2), CREB1, PAI2, AKT(PKB), NF-kB p50/p65, Gastrin 17, PDK (PDPK1), c-Src, TAK1(MAP3K7), MEK1(MAP2K1), PI3K cat class IA, SOS, MEF2, MEK4(MAP2K4), ELAVL1 (HuR), MEF2D, MEKK1(MAP3K1), PKC-alpha, IKK-beta, FAK1, G-protein alpha-q, JNK(MAPK8-10), Elk-1
34	Apoptosis and survival_BAD phosphorylation	2.12E-08	Calcineurin A (catalytic), IRS-1, H-Ras, 14-3-3, PKA-reg (cAMP-dependent), PP2C, Cytochrome c, CDK1 (p34), BAD, Bax, PI3K reg class IA, PP1-cat alpha, MEK2(MAP2K2), IGF-1 receptor, G-protein beta/gamma, AKT(PKB), PDK (PDPK1), MEK1(MAP2K1), PI3K cat class IA, Adenylate cyclase type I, SOS, PP2A catalytic, p90Rsk, p70 S6 kinase1, G-protein alpha-s, Bcl-2, Beclin 1, p70 S6 kinase2
35	Inhibition of apoptosis in gastric cancer	2.12E-08	Gastrin 17-Gly, TGF-beta 1, DR5(TNFRSF10B), Apaf-1, Apo-2L(TNFSF10), Cytochrome c, Caspase-8, BAD, Bax, Caspase-3, NF-kB, AKT(PKB), DR4(TNFRSF10A), Gastrin 17, Progastrin, Mcl-1, TGF-beta receptor type II, PAK1, ROCK, Caspase-7, Gasdermin, tBid, Bcl-2, HtrA2, HGF receptor (Met), Smac/Diablo, Bim, Bid
36	Apoptosis and survival_Endoplasmic reticulum stress response pathway	2.3E-08	p38alpha (MAPK14), GRP78, C/EBP zeta, Apaf-1, PP1-cat, ATF-4, S2P, GADD34, ATF-6 alpha (90kDa), Bak, Cytochrome c, I-kB, Bax, PP1-cat alpha, eIF2S1, Calpain 1(mu), NF-kB p50/p65, ATF-6 alpha (50kDa), Derlin-2, eIF2AK3, ASK1 (MAP3K5), MEK3(MAP2K3), S1P, MEK4(MAP2K4), Caspase-7, Caspase-12, tBid, Bcl-2, IP3R1, JNK(MAPK8-10), DNAJC3, Bim, Bid, ERP5
37	Development_Positive regulation of STK3/4 (Hippo) pathway and negative regulation of YAP/TAZ function	2.71E-08	Willin, Casein kinase I delta, SCRIB, Cullin 2, INADL, AMPK beta subunit, Alpha-1 catenin, 14-3-3, Casein kinase I epsilon, AMPK gamma subunit, Beta-catenin, PKA-reg (cAMP-dependent), ZO-2, RASSF2, CRB3, STK4, Adenylate cyclase, Itch, RASSF5, LATS2, Actin cytoskeletal, Skp2/TrCP/FBXW, TAZ, LRR-1, AMOTL1 (Jeap), Axin, beta-TrCP, AMPK alpha subunit, EBP50, MARKK, Alpha-catenin, MALS-3, Cul2/Rbx1 E3 ligase, G-protein alpha-s, FasR(CD95), Mol1b, Beta-2 adrenergic receptor, PEZ, Angiomotin (AMOT), LIF receptor
38	Development_FGFR signaling pathway	2.95E-08	GAB1, H-Ras, Syndecan-4, FGFR1, Rac1, IP3 receptor, Ubiquitin, MAPKAPK2, EPS8, PI3K reg class IA, PLC-gamma 1, MEK6(MAP2K6), MEK2(MAP2K2), CREB1, AKT(PKB),

			PDK (PDPK1), E3b1(ABI-1), MEK1(MAP2K1), PI3K cat class IA, Syndecan-1, DOCK1, FGF2, SOS, PAK1, MEK4(MAP2K4), MEKK1(MAP3K1), SHP-2, Perlecan, JNK(MAPK8-10), SHB, p38 MAPK, Elk-1, Syndecan-2
39	Neurophysiological process_Dynein-dynactin motor complex in axonal transport in neurons	2.95E-08	CDK5, NudE, Importin (karyopherin)-alpha, Ubiquitin, DYNLL, ORP1, Dynein 1, cytoplasmic, heavy chain, Tctex-1, MAPRE3(EB3), DYNLT, HAP40, AKT(PKB), Snapin, PAFAH alpha (LIS1), DYI2, NT-3, Tubulin (in microtubules), Importin (karyopherin)-beta, Dynein 1, cytoplasmic, light chains, TrkC, Sortilin, BPAG1, TrkB, JSAP1, Rab-5A, Kinesin heavy chain, Bassoon, Kinesin light chain, Carboxypeptidase H, Dynein 1, cytoplasmic, intermediate chains, BDNF, NUDEL, SPTBN2
40	DNA damage_ATM/ATR regulation of G2/M checkpoint: nuclear signaling	3.73E-08	NFBD1, Cyclin B, Cyclin B2, PALB2, CDK1 (p34), SMAR1, Chk1, Wee1, Cyclin B1, Mcl-1, PLK1, Chk2, Brca1, CDC25C, DNA-PK, Histone H2AX, BTG2, ATR, PCBP-4 (mcg10), CDC14b, ATRIP, CDK2, CDC18L (CDC6), HSF1, 14-3-3 sigma, Ku70, Cyclin A, DNMT1, TTK
41	Cytoskeleton remodeling_FAK signaling	4.32E-08	Cyclin D3, H-Ras, VEGFR-2, G-protein alpha-q/11, Fibronectin, FARP2, Rac1, IP3 receptor, Talin, GRP-R, GSK3 beta, PI3K reg class IA, PLC-beta, PLC-gamma 1, RIPK1, MEK2(MAP2K2), CDC42, AKT(PKB), c-Src, MEK1(MAP2K1), PI3K cat class IA, TRAF3, DOCK1, SOS, PAK1, C3G, MKK7 (MAP2K7), p130CAS, Calmodulin, PTEN, GRP(1-27), MEKK1(MAP3K1), FAK1, Elk-1
42	Cell cycle_Role of Nek in cell cycle regulation	4.49E-08	Nek2A, IRS-1, MAD2a, RCC1, Nek11, CDK1 (p34), PI3K reg class IA, NEK7, Cyclin B1, TPX2, PDK (PDPK1), Ran, Tubulin (in microtubules), Tubulin beta, Tubulin gamma, PI3K cat class IA, Histone H3, Aurora-A, p70 S6 kinase1, Tubulin alpha, NEK1, HEC, Histone H1
43	Cytoskeleton remodeling_Reverse signaling by Ephrin-B	4.49E-08	F-Actin, H-Ras, Beta-catenin, GSK3 beta, NCK2, G-protein beta/gamma, Actin cytoskeletal, Tau (MAPT), c-Src, Axin, SDF-1, CXCR4, WaspIP, Tubulin (in microtubules), PINCH, G-protein alpha-i family, SOS, PAK1, ILK, Tubulin alpha, Ephrin-B, FAK1, Ephrin-B receptors
44	Signal transduction_Calcium-mediated signaling	4.62E-08	Calcineurin A (catalytic), c-Fos, 14-3-3, CaMK I, EGR1, MUNC13, p47-phox, Rac1, IP3 receptor, BAD, MLCP (reg), I-kB, MEK6(MAP2K6), CREB1, PPARGC1 (PGC1-alpha), NF-kB, AKT(PKB), NF-AT2(NFATC1), MLCP (cat), Tiam1, cPKC (conventional), RelA (p65 NF-kB subunit), ASK1 (MAP3K5), MEK3(MAP2K3), AMPK alpha subunit, Myosin II, MEF2, p300, MEK4(MAP2K4), ROCK, Calmodulin, PKC, MYH11, PKC-alpha, IKK-beta, JNK(MAPK8-10), Bcl-10, NURR1, p38 MAPK, Elk-1
45	Ligand-independent activation of Androgen receptor in Prostate Cancer	4.72E-08	STAT5A, FGFR2, GAB1, WNT3A, IRS-1, Neuregulin 1, H-Ras, Beta-catenin, PP2A regulatory, STAT5B, FGFR1, Kallikrein 3 (PSA), Prolactin receptor, Androgen receptor, GSK3 beta, PI3K reg class IA, N-Ras, MEK2(MAP2K2), IGF-1 receptor, AKT(PKB), PDK (PDPK1), MEK1(MAP2K1), PI3K cat class IA,

			B-Raf, K-RAS, NCOA3 (pCIP/SRC3), Tcf(Lef), FGF2, SOS, PP2A catalytic, NCOA2 (GRIP1/TIF2), FRS2beta, c-Abl, Cyclin D1, NCOA1 (SRC1), HDAC1, SRD5A1, Frizzled
46	Development_Thrombopoietin signaling via ERK1/2 and PI3K	4.72E-08	PDK1, Cyclin D3, c-Fos, GAB1, IRS-2, BMP4, PP1-cat, H-Ras, NF-kB p50/c-Rel, PKC-zeta, CrkL, EGR1, Thrombopoietin, PI3K reg class IA (p85), GSK3 beta, CREB1, MEK1/2, AKT(PKB), PDK (PDPK1), p27KIP1, GAB2, PI3K cat class IA, RelA (p65 NF-kB subunit), B-Raf, ITGA2B, HIF1A, DNA-PK, PDHA (somatic), SOS1, C3G, AML1 (RUNX1), 4E-BP1, p70 S6 kinase1, SHP-2, FOXO3A, SP1, p38 MAPK, Elk-1
47	TGF-beta 1-induced transactivation of membrane receptors signaling in HCC	4.79E-08	TGF-beta 1, alpha-6/beta-1 integrin, Beta-catenin, Fibronectin, Actin, Rac1, GSK3 beta, PI3K reg class IA, CDK4, AKT(PKB), PDGF receptor, c-Src, Axin, alpha-3/beta-1 integrin, ITGB1, PI3K cat class IA, TGF-beta receptor type II, DOCK1, TGF-beta, ITGA6, PAK1, CDK2, p130CAS, PTEN, Cyclin E, SLUG, FAK1, c-Abl, Cyclin A, Cyclin D1, PDGF-R-beta
48	Development_VEGF signaling via VEGFR2 - generic cascades	5.03E-08	Calcineurin A (catalytic), NCK1, H-Ras, TCF7L2 (TCF4), ROCK1, eIF4E, Beta-catenin, VEGFR-2, Rac1, IP3 receptor, I-kB, p120GAP, GSK3 beta, MAPKAPK2, PI3K reg class IA, PLC-gamma 1, MEK2(MAP2K2), CREB1, PLAUR (uPAR), CDC42, Actin cytoskeletal, Fyn, MSK1, AKT(PKB), HSP90, NF-kB p50/p65, PDK (PDPK1), c-Src, TSAD, NF-AT2(NFATC1), MLCK, MEK1(MAP2K1), PI3K cat class IA, MEK3(MAP2K3), SOS, PAK1, p90Rsk, Calmodulin, PKC, MEKK1(MAP3K1), Vinculin, PKC-alpha, HSP27, IKK-beta, FAK1, Neurofibromin, SHB, p38 MAPK
49	Cell cycle_Initiation of mitosis	5.28E-08	Cyclin B2, CDK1 (p34), CDK7, Wee1, Lamin B, AKT(PKB), Cyclin B1, PLK1, Histone H3, CDC25C, CDC25B, MYRL2, FOXM1, p90RSK1, AP-2A, Cyclin H, Nucleolin, Kinase MYT1, KNSL1, Histone H1
50	Main pathways of Schwann cells transformation in neurofibromatosis type 1	5.37E-08	PDGF-B, RHEB2, c-Fos, Neuregulin 1, H-Ras, Beta-catenin, IP3 receptor, BAD, PI3K reg class IA (p85), Amphiregulin, GSK3 beta, PLC-gamma 1, N-Ras, IGF-1 receptor, MEK1/2, CDC42, AKT(PKB), PDGF receptor, PDK (PDPK1), SOX9, SDF-1, CXCR4, MEK1(MAP2K1), ITGB1, PI3K cat class IA, K-RAS, HIF1A, S100B, BRD4, ErbB4, PAK1, Myelin basic protein, p70 S6 kinase1, Calmodulin, PTEN, Bcl-2, FAK1, Neurofibromin, Cyclin D1, p38 MAPK, Bim, Elk-1, PDGF-R-beta
